# Epidemiology and determinants of non-diabetic hyperglycaemia and its conversion to type 2 diabetes mellitus, 2000–2015: cohort population study using UK electronic health records

**DOI:** 10.1136/bmjopen-2020-040201

**Published:** 2020-09-06

**Authors:** Rathi Ravindrarajah, David Reeves, Elizabeth Howarth, Rachel Meacock, Claudia Soiland-Reyes, Sarah Cotterill, William Whittaker, Simon Heller, Matt Sutton, Peter Bower, Evangelos Kontopantelis

**Affiliations:** 1 Division of Population Health, Faculty of Biology, Medicine and Health, The University of Manchester, Manchester, UK; 2 Research & Innovation, Northern Care Alliance NHS Group, Summerfield House, M5 5AP, Salford, Salford, UK; 3 Centre for Biostatistics, University of Manchester, Manchester, UK; 4 Academic Unit of Diabetes, Endo and Metab, Unversity of Sheffield, Sheffield, UK

**Keywords:** diabetes & endocrinology, epidemiology, primary care

## Abstract

**Objectives:**

To study the characteristics of UK individuals identified with non-diabetic hyperglycaemia (NDH) and their conversion rates to type 2 diabetes mellitus (T2DM) from 2000 to 2015, using the Clinical Practice Research Datalink.

**Design:**

Cohort study.

**Settings:**

UK primary Care Practices.

**Participants:**

Electronic health records identified 14 272 participants with NDH, from 2000 to 2015.

**Primary and secondary outcome measures:**

Baseline characteristics and conversion trends from NDH to T2DM were explored. Cox proportional hazards models evaluated predictors of conversion.

**Results:**

Crude conversion was 4% within 6 months of NDH diagnosis, 7% annually, 13% within 2 years, 17% within 3 years and 23% within 5 years. However, 1-year conversion fell from 8% in 2000 to 4% in 2014. Individuals aged 45–54 were at the highest risk of developing T2DM (HR 1.20, 95% CI 1.15 to 1.25— compared with those aged 18–44), and the risk reduced with older age. A body mass index (BMI) above 30 kg/m^2^ was strongly associated with conversion (HR 2.02, 95% CI 1.92 to 2.13—compared with those with a normal BMI). Depression (HR 1.10, 95% CI 1.07 to 1.13), smoking (HR 1.07, 95% CI 1.03 to 1.11—compared with non-smokers) or residing in the most deprived areas (HR 1.17, 95% CI 1.11 to 1.24—compared with residents of the most affluent areas) was modestly associated with conversion.

**Conclusion:**

Although the rate of conversion from NDH to T2DM fell between 2010 and 2015, this is likely due to changes over time in the cut-off points for defining NDH, and more people of lower diabetes risk being diagnosed with NDH over time. People aged 45–54, smokers, depressed, with high BMI and more deprived are at increased risk of conversion to T2DM.

**Strengths and limitations of this study**

- Data were based on a large, anonymised, longitudinal and nationally representative sample of general practices.
- The length of the study period (2000–2015) was useful in capturing changes over time.
- Cases of non-diabetic hyperglycaemia (NDH) and type 2 diabetes mellitus were identified using Read codes, and the quality of recording may have been problematic for the former in earlier years.
- Our NDH code list included a few relevant items and is not sensitive to misclassification.

## Introduction

The proportion of the population with type 2 diabetes mellitus (T2DM) has been rising globally and is an important contributor to mortality, morbidity and healthcare costs. It has been estimated that 415 million people live with diabetes across the globe and 193 million people have undiagnosed diabetes.^1^ It has been suggested that currently there are 5 million people in England who are at risk of developing T2DM.^2^ T2DM is characterised by pancreatic dysfunction causing insulin resistance. There are other key pathophysiological processes which increase the risk of T2DM, which involves organs including pancreas, liver, skeletal muscle, kidneys, brain, small intestine and adipose tissue.^3^ Lifestyle factors such as excess weight and physical inactivity are known to increase the risk of developing T2DM.

Non-diabetic hyperglycaemia (NDH) also known as pre-diabetes or impaired glucose regulation, IGR), refers to levels of blood glucose that are increased from the normal range but not yet high enough to be in the diabetic range. Previous research has shown that individuals diagnosed with NDH are at a higher risk of developing T2DM.^4^ The NHS RightCare diabetes pathway defines NDH as having an HbA1c(haemoglobin A1c or glycated haemoglobin) measurement in the 42–47 mmol/mol range (6.0%–6.4%), or fasting plasma glucose in the 5.5–6.9 mmol/mol range.^5^ Previous analyses using Health Survey England data have shown discrepancies in the prevalence of NDH in the UK. While one study suggested that the average NDH prevalence was 11% in adults aged 16+ in England, in the period between 2009 and 2013,^6^ the other suggested a sharp rise in the prevalence of NDH from 11.6% in 2003 to 35.3% in 2011 in all adults.^7^ The use of different cut-points for HbA1C used to define NDH has been suggested as the cause of this discrepancy; one study used the National Institute for Health and Care Excellence (NICE) and Diabetes UK cut-points (HbA1C: 42–47 mmol/mol) whereas the other used the American Diabetes Association cut-points (HbA1C: 39–47 mmol/mol). Delaying or preventing T2DM has become an international priority due to the burden that the condition places on both patients and health services.^8^ NHS England, Public Health England and Diabetes UK have implemented a programme to identify those at high risk of developing T2DM and offer them an evidence-based behavioural intervention (National health Service Diabetes Prevention Programme) to people identified as having NDH in an attempt to reduce the incidence of T2DM and the complications related to it.^9^

This paper explores two aspects of the epidemiology of people diagnosed with NDH in UK primary care. First, we aimed to estimate the prevalence of NDH and to explore the characteristics of patients with NDH in a population cohort of adults from 2000 to 2015. We chose this study period both to ensure high-quality data and to avoid introducing bias into our analysis from any potential effects from the National Diabetes Prevention Programme.^10^ Second, we evaluated the conversion rates of NDH to T2DM over time, and whether conversion rates differ by age, sex, body mass index (BMI) levels, depression, multimorbidity and area-level deprivation.

## Methods

### Data source

Patient-level data were obtained from the Clinical Practice Research Datalink (CPRD), one of the largest active primary care databases of electronic health records (EHR) in the UK.^11^ This dataset captures approximately 7% of the total UK population. The database holds anonymised data which contains information on clinical signs, diagnoses, tests and procedures.^11^ Approximately 60% of all UK CPRD practices participate in the CPRD linkage scheme, which provides additional patient-level information. For this work, we obtained patient-level deprivation through the Office of National Statistics linkage, in the form of the 2010 Index of Multiple Deprivation (IMD).^12^

### Study participants

Practices taking part in the CPRD are checked for eligibility in each year using a CPRD assessment algorithm, and evaluated to be of research standard or not. Patients were regarded as eligible if they had been registered with a practice for a full year, were aged 18 years and over and had a code for NDH between 1 April 2000 and 31 March 2016. At least one relevant Read code was considered adequate to flag a patient. Codes were identified using a strategy that involved searching for relevant terms through an algorithm, with the returned list being reviewed and finalised by members of the research team, as described elsewhere.^13 14^ Read codes which were actively used by general practitioners (GPs) to identify NDH were included in the study: 44v2.00 (Glucose Tolerance Test impaired), C11y200 (Impaired glucose tolerance, IGT), C11y300 (Impaired fasting glycaemia), C11y500 (pre-diabetes), C317.00 (NDH), R102.00 ((D) Glucose Tolerance Test abnormal), R102.11 ((D) Pre-diabetes), R102.12 ((D) IGT test), R10D000 ((D) Impaired fasting glycaemia), R10D011 ((D) Impaired fasting glucose, IFG), R10E.00 ((D) IGT. Eligible patients were followed up until censored at the earliest of any of the following events: diagnosed with T2DM (the outcome event), transferred out of practice (any cause), last collection date for the practice, end date of the study (31 March 2016) or death. To report prevalence, we also included cases that were diagnosed with NDH at any point prior to 1 April 2000, who met all other inclusion criteria.

### Study measures

We calculated the prevalence of NDH in each year between 2000 and 2015, and conversion to T2DM was also determined. People with at least one relevant Read code of T2DM following the NDH diagnosis (the index date), were considered to have progressed to T2DM during the study period (online supplementary table 1 provides a list of read codes used to diagnose T2DM). Patients with a previous record of type 1 diabetes were excluded.

10.1136/bmjopen-2020-040201.supp1 Supplementary data

We extracted information on the following covariates which have previously been reported^10^ to be relevant to NDH and T2DM; age, gender, BMI, total serum cholesterol, smoking status, socioeconomic status and depression. Age was grouped into the following bands: 18–44, 45–54, 55–64, 65–74, 75–84 and 85 years or over. The latest available measurement before the NDH diagnosis date, up until the previous 12 months, was used to define baseline total cholesterol and BMI. If such a value was not available, the measurement was set to missing. BMI values were classified into the following categories: underweight (<18.5 kg/m^2^), normal weight (18.5–24.9 kg/m^2^), overweight (25.0–29.9 kg/m^2^) and obese (≥30 kg/m^2^). Total serum cholesterol in mmol/L was categorised into: under 3.0, (3.0, 4.0), (4.0, 5.0), (5.0, 6.0) and 6.0 or over. We also quantified the multimorbidity burden, using the Charlson Comorbidity Index (CCI), which is a widely used measure which assigns different weights to different conditions and includes: any malignancy, cerebrovascular disease, chronic pulmonary disease, congestive cardiac disease, dementia, HIV/AIDS, hemiplegia, lymphoproliferative disorders, metastatic solid tumour, mild liver disease, moderate and severe liver disease (CCI also includes diabetes with complications, which we necessarily excluded).^15 16^ This modified CCI was calculated using the list of validated diagnostic primary care Read codes used by Khan *et al*.^15^ Participants were classified as having a condition if the condition was present at diagnosis of NDH or 12 months prior to diagnosis of NDH. CCI takes integer values and was categorised as: 0, 1–2, 3–4 and >4. Depression was evaluated using medical codes and therapy codes which were obtained from the code lists derived from the CPRD provided on a Cambridge University repository.^17^ Participants were considered to have depression at the index date (the date of NDH diagnosis) if they were recorded as depressed either by a code or if they were on relevant medication in the last 12 months. Smoking status was determined from information in the patients’ record and categorised as ‘smoker’, ‘ex-smoker’ or ‘never smoked’. The IMD was used to classify deprivation and the IMD scores were divided into quintiles.

### Conversion of NDH to T2DM

**Figure 1:**
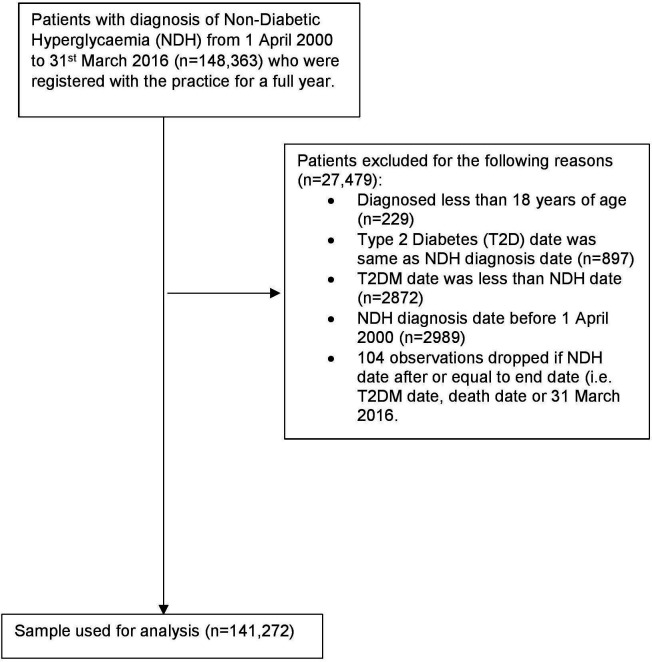
Flow diagram of patient inclusions. T2DM, type 2 diabetes mellitus.

The time of conversion of NDH to T2DM was defined as the time from the index date (diagnosis of NDH) to the date they were diagnosed as having T2DM. This time was then categorised into progression time of: 1 month; 3 months, 6 months, 12 months, 2 years, 3 years, 4 years and 5 years. Those who had a conversion time of over 5 years were excluded from analysis. In addition, patients who did not convert to T2DM, left the study or died within this study period were categorised into a single category as ‘Not converted/left/died’. A small number of participants were diagnosed as having T2DM on, or ever before, the index date, and were excluded from further analyses (See figure 1).

### Statistical analysis

The characteristics of people identified with NDH are presented descriptively. Conversion rates of NDH to T2DM, in the progression time categories were plotted over time. Annual bins were defined as financial years, for example 1 April 2000 to 31 March 2001 was labelled as 2000. The associations between covariates and conversion from NDH to T2DM were estimated in a time to event analysis. A Cox proportional hazards model was employed to estimate adjusted HRs of the associations between conversion and the following covariates: gender, age groups, BMI categories, total cholesterol levels, depression, year, patient-level deprivation scores and CCI categories. Proportionality of hazards was tested using Schoenfeld residuals.

### Patient involvement

CPRD data provide anonymised patient data, hence patients are not identified by the researchers.

## Results

**Figure 2:**
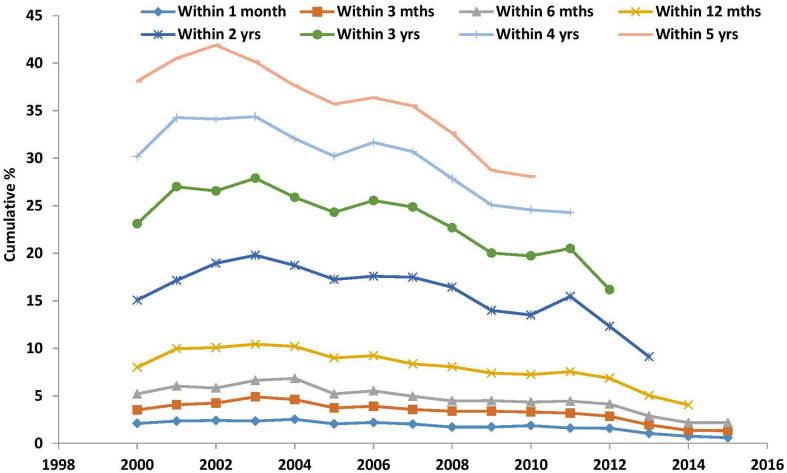
Cumulative conversion of NDH to T2DM diabetes from 2000 to 2015. Year 2000 defined as 1 April 2000 till 31 st March 2001 and other years defined similarly. NDH, non-diabetic hyperglycaemia; T2DM, type 2 diabetes mellitus.

Over the study period, a total of 148 363 participants were identified with NDH. The prevalence and incidence of NDH for each financial year is shown in table 1. Prevalence increased from 0.07% in 2000 to 1.85% in 2015. Incidence of NDH increased from 0.02% in 2000 to 0.21% in 2015. Table 2 and figure 2 show the cumulative frequency of conversion from NDH to T2DM, by year, from 1 April 2000 to 31 March 2016. Frequency of conversion within one financial year peaks in 2003 and then follows a decreasing trend. Among this general trend of declining conversion, there was a peak in the year 2011, with a further exploration of the data (results not shown) suggesting that patients had somewhat higher BMIs in this year, although that does not fully explain the rise.

**Table 1:** Prevalence and Incidence of NDH

	Prevalence			Incidence		
**Year**	**Numerator**	**Denominator**	**%**	**Numerator**	**Denominator**	**%**
2000	2809	3 784 862	0.07	750	3 782 803	0.02
2001	4065	3 825 769	0.11	1256	3 822 960	0.03
2002	6627	3 868 575	0.17	2562	3 864 510	0.07
2003	10 790	3 905 077	0.28	4163	3 898 450	0.11
2004	16 687	3 957 556	0.42	5897	3 946 766	0.15
2005	23 989	3 996 114	0.60	7302	3 979 427	0.18
2006	29 805	4 029 795	0.74	5816	4 005 806	0.15
2007	35 730	4 074 123	0.88	5925	4 044 318	0.15
2008	41 930	4 130 943	1.02	6200	4 095 213	0.15
2009	48 116	4 191 018	1.15	6186	4 149 088	0.15
2010	52 891	4 245 410	1.25	4775	4 197 294	0.11
2011	57 556	4 283 200	1.34	4665	4 230 309	0.11
2012	61 787	4 335 322	1.43	4231	4 277 766	0.10
2013	68 376	4 383 749	1.56	6589	4 321 962	0.15
2014	74 423	4 446 718	1.67	6047	4 378 342	0.14
2015	83 652	4 528 613	1.85	9229	4 454 190	0.21

Year 2000 defined as 1 April 2000 till 31 March 2001 and other years defined similarly.

NDH, non-diabetic hyperglycaemia.

**Table 2:** Cumulative frequency of conversion from NDH to T2DM from 2000 to 2015

Year	Within 1 month				Within 3 months				Within 6 months				Within 1 year			
	N remaining uncon verted	N con verted	N censored	Cum % con verted	N remaining uncon verted	N con verted	N censored	Cum % con verted	N remaining uncon verted	N con verted	N censored	Cum % con verted	N remaining uncon verted	N con verted	N censored	Cum % con verted
2000	887	19	1	2.1	870	13	4	3.53	854	15	1	5.2	818	25	11	7.99
2001	1460	35	0	2.34	1433	26	1	4.08	1397	29	7	6.03	1320	58	19	9.96
2002	2922	72	2	2.4	2863	55	4	4.24	2803	47	13	5.82	2650	126	27	10.07
2003	4793	115	5	2.34	4655	125	13	4.89	4538	85	32	6.63	4276	183	79	10.43
2004	7076	184	6	2.53	6907	151	18	4.62	6698	160	49	6.83	6370	241	87	10.21
2005	8832	185	7	2.05	8660	152	20	3.74	8479	132	49	5.21	8007	335	137	8.99
2006	8561	193	4	2.2	8389	149	23	3.91	8194	140	55	5.52	7743	319	132	9.23
2007	9240	192	14	2.03	9073	144	23	3.56	8912	130	31	4.95	8472	317	123	8.35
2008	10 243	179	10	1.72	10 046	172	25	3.37	9871	114	61	4.47	9391	370	110	8.07
2009	10 923	191	8	1.72	10 721	185	17	3.38	10 553	123	45	4.49	10 100	319	134	7.4
2010	9991	189	4	1.86	9828	146	17	3.29	9686	107	35	4.35	9279	291	116	7.24
2011	9973	163	6	1.61	9792	161	20	3.2	9628	126	38	4.45	9181	309	138	7.53
2012	10 057	162	5	1.58	9912	130	15	2.86	9743	131	38	4.14	9366	274	103	6.85
2013	12 267	131	17	1.06	12 130	110	27	1.94	11 963	115	52	2.88	11 537	264	162	5.03
2014	11 318	85	14	0.74	11 214	71	33	1.37	11 061	92	61	2.18	10 717	209	135	4.04
2015	12 832	81	1080	0.6	10 111	85	2636	1.34	6716	72	3323	2.18				
	**Within 2 years**				**Within 3 years**				**Within 4 years**				**Within 5 years**			
2000	734	62	22	15.06	634	68	32	23.1	545	57	32	30.2	456	60	29	38.09
2001	1160	103	57	17.14	971	135	54	27.01	827	94	50	34.26	694	76	57	40.52
2002	2283	256	111	18.95	1973	210	100	26.57	1674	198	101	34.13	1377	191	106	41.89
2003	3647	437	192	19.8	3105	359	183	27.89	2672	272	161	34.38	2305	228	139	40.13
2004	5490	590	290	18.72	4726	471	293	25.88	4086	384	256	32.07	3533	325	228	37.63
2005	6939	711	357	17.25	6025	577	337	24.3	5275	459	291	30.21	4650	406	219	35.7
2006	6741	700	302	17.6	5841	638	262	25.55	5076	467	298	31.66	4468	341	267	36.37
2007	7328	829	315	17.49	6385	643	300	24.88	5612	484	289	30.71	4959	379	274	35.5
2008	8176	836	379	16.42	7247	602	327	22.7	6473	474	300	27.86	5763	421	289	32.66
2009	9059	708	333	14	8049	621	389	20.02	7229	500	320	25.09	6597	344	288	28.73
2010	8324	616	339	13.51	7427	587	310	19.73	6712	440	275	24.57	6186	306	220	28.07
2011	8091	773	317	15.46	7303	473	315	20.5	6703	342	258	24.29	0	137	6566	27.32
2012	8467	537	362	12.30	7769	366	332	16.17								
2013	10625	487	425	9.12												

NDH, non-diabetic hyperglycaemia; T2DM, type 2 diabetes mellitus.

After all exclusion criteria were applied (see figure 1), our final NDH population was 141 272 people, with a mean follow-up period of 5 years since the index date.

Table 3 displays the baseline characteristics of the cohort. Covariates are treated as categorical variables in our analysis, and so reported here as numbers and percentages. The mean age of the cohort was 63.2 (SD=13.4) years, and 52% were male. The prevalence of NDH was highest in those aged 65–74 years (39 178/141 272; 27.7%). The proportion of NDH was higher in older females (3728/67 369, 5.5%), compared with older males (2162/73 903; 2.9%) aged 85 years and more. The most common BMI category in our cohort was obese, with 32% of females with a measurement of BMI equal to or above 30 kg/m^2^. Results showed that 19% of the NDH cohort had depression when they were diagnosed with NDH. The vast majority of the NDH population (85%) had a Charlson comorbidity score of 0 at the index date, indicating absence of major comorbidities.

**Table 3:** Characteristics of the cohort

	All	Males	Females
N	141 272	73 903 (52.3)	67 369 (47.7)
Age (years)	63.2±13.4	62.8±12.4	63.6±14.5
Age group count (%)			
18–44	12 896 (9.1)	5619 (7.6)	7277 (10.8)
45–54	22 717 (16.1)	12 934 (17.5)	9783 (14.5)
55–64	36 790 (26.0)	21 127 (28.6)	15 663 (23.3)
65–74	39 178 (27.7)	21 042 (28.5)	18 136 (26.9)
75–84	23 801 (16.9)	11 019 (14.9)	12 782 (19.0)
≥85	5890 (4.2)	2162 (2.9)	3728 (5.5)
Smoking status count (%)			
Current	21 088 (14.9)	11 352 (15.4)	9736 (14.5)
Ex	46 301 (32.8)	27 979 (37.9)	18 322 (27.2)
Never	27 834 (19.7)	12 046 (16.3)	15 788 (23.4)
Missing	46 049 (32.6)	22 526 (30.5)	23 523 (34.9)
BMI categories (kg/m^2^) count (%)			
<18.5	628 (0.4)	153 (0.2)	475 (0.7)
18.5–25	11 553 (8.2)	5504 (7.5)	6049 (9.0)
25–30	27 523 (19.5)	16 686 (22.6)	10 837 (16.1)
≥30	42 456 (30.1)	21 189 (28.7)	21 267 (31.6)
Missing	59 112 (41.8)	30 371 (41.1)	28 741 (42.7)
Cholesterol (%) count (%)			
<3	1538 (1.1)	1203 (1.6)	336 (0.5)
3–4	12 668 (9.0)	8814 (11.9)	3859 (5.7)
4–5	29 204 (20.7)	17 170 (23.2)	12 041 (17.9)
5–6	28 554 (20.2)	14 889 (20.1)	13 670 (20.3)
≥6	22 818 (16.2)	9844 (13.3)	12 979 (19.3)
Missing	46 490 (32.9)	22 002 (29.8)	24 513 (36.4)
Depression	26 064 (18.5)	9724 (13.2)	16 340 (24.3)
CCI score count (%)			
None	120 158 (85.1)	63 571 (86.0)	56 587 (84.0)
1–2	20 912 (14.8)	10 215 (13.8)	10 697 (15.9)
3–4	142 (0.1)	85 (0.1)	57 (0.1)
>4	60 (0.04)	32 (0.04)	28 (0.04)
Patient-level deprivation index (2010 IMD score) count (%)			
Quintile 1(most affluent)	12 854 (9.1)	7034 (9.5)	5820 (8.6)
Quintile 2	13 617 (9.6)	7368 (10.0)	6249 (9.3)
Quintile 3	12 882 (9.1)	6692 (9.1)	6190 (9.2)
Quintile 4	12 816 (9.1)	6514 (8.8)	6302 (9.4)
Quintile 5 (least affluent)	9866 (7.0)	4780 (6.5)	5086 (7.6)
Missing	79 237 (56.1)	41 515 (56.2)	37 722 (56.0)

BMI, body mass index; CCI, Charlson Comorbidity Index; IMD, Index of Multiple Deprivation.

Table 4 shows the number of patients who converted from NDH to T2DM. Over the whole of the study period, the conversion rates were: 1.6% within 1 month, 3% within 3 months, 4.2% within 6 months, 7% within a year, 12.8% within 24 months, 17.2% within 3 years, 20.4% within 4 years and 22.8% over 5 years. The majority (77.2%, n=104 030) did not convert, but the length of time each was followed up varied depending on the time they were diagnosed with NDH.

**Table 4:** Conversion from at risk of diabetes (NDH) to T2DM

Time taken to convert from at risk to type 2 diabetes (T2D)	Numerator (total number diagnosed with T2D)	Denominator (total number with NDH)	%	% Change
Within 1 month	2176	134 734	1.62	
Within 3 months	4051	134 734	3.01	1.39
Within 6 months	5669	134 734	4.21	1.20
Within 1 year	9369	134 734	6.95	2.75
Within 2 years	17 216	134 734	12.78	5.82
Within 3 years	23 168	134 734	17.20	4.42
Within 4 years	27 490	134 734	20.40	3.21
Within 5 years	30 704	134 734	22.79	2.39

NDH, non-diabetic hyperglycaemia; T2DM, type 2 diabetes mellitus.

Table 5 shows the results from the Cox proportional hazard models, which explored time to conversion from NDH to T2DM, with failure being the diagnosis of T2DM. Residuals were linear over time, indicating that proportionality generally stood. The rate of conversion was highest for the 45–54 age group with HR 1.20 (95% CI 1.15 to 1.25), compared with those aged 18–44, and the risk steadily decreased with increasing age to an HR 0.65 (95% CI 0.60 to 0.71) for people aged 85 or over. Cholesterol categories did not appear to be strongly associated with conversion to T2DM. People with high BMI had a much higher risk of conversion to T2DM, with those classed overweight (BMI 25–30) having an HR 1.40 (95% CI 1.33 to 1.48), and those classed obese (BMI≥30) having an HR 2.0 (95% CI 1.9 to 2.1), compared with individuals with a normal BMI (18.5–25 kg/m^2^). Compared with non-smokers, current smokers had a slightly increased risk of converting to T2DM with an HR 1.07 (95% CI of 1.03 to 1.11). Those who had a CCI score of 1–2 had a slightly higher risk of conversion to T2DM with an HR 1.1 (95% CI 1.08 to 1.15) but there was no increased risk among those with higher CCI scores. Having depression at baseline slightly increased the risk of conversion (HR 1.10, 95% CI 1.07 to 1.13). The risk of conversion to T2DM increased with patient-level deprivation as measured by the 2010 IMD, suggesting that those living in more deprived areas are at an increased risk of conversion from NDH to T2DM. Patients living in the least affluent quintile had an HR 1.17 (95% CI 1.11 to 1.24), compared with patients living in the most affluent quintile.

**Table 5:** Cox proportional hazard models exploring time to conversion from NDH to T2DM for patients by baseline characteristics

	HR (95% CI)	P value
Males	Ref	
Females	0.97 (0.95 to 0.99)	0.009
Age group (years)		
18–44	Ref	
45–54	1.20 (1.15 to 1.25)	<0.001
55–64	1.10 (1.06 to 1.14)	<0.001
65–74	1.03 (0.99 to 1.07)	0.13
75–84	0.86 (0.82 to 0.90)	<0.001
≥85	0.65 (0.60 to 0.71)	<0.001
Cholesterol categories (%)		
<3	1.04 (0.95 to 1.16)	0.391
3–4	1.03 (0.99 to 1.07)	0.165
4–5	Ref	
5–6	0.94 (0.92 to 0.98)	0.001
≥6	0.92 (0.89 to 0.95)	<0.001
Missing	0.91 (0.89 to 0.94)	<0.001
Smoking status		
Non smoker	Ref	
Current smoker	1.07 (1.03 to 1.11)	<0.001
Ex- smoker	0.98 (0.96 to 1.01)	0.312
missing	0.98 (0.95 to 1.02)	0.338
BMI categories (kg/m^2^)		
<18.5	0.59 (0.44 to 0.78)	<0.001
18.5–25	Ref	
25–30	1.40 (1.33 to 1.48)	<0.001
≥30	2.02 (1.92 to 2.13)	<0.001
Missing	1.44 (1.37 to 1.52)	<0.001
Depression	1.10 (1.07 to 1.13)	<0.001
CCI Score		
None	Ref	
1–2	1.11 (1.08 to 1.15)	<0.001
3–4	0.98 (0.68 to 1.43)	0.934
>4	1.67 (0.99 to 2.81)	0.057
Patient-level deprivation index		
Quintile 1(most affluent)	Ref	
Quintile 2	1.08 (1.03 to 1.13)	0.002
Quintile 3	1.03 (0.98 to 1.08)	0.237
Quintile 4	1.12 (1.07 to 1.18)	<0.001
Quintile 5 (least affluent)	1.17 (1.11 to 1.24)	<0.001
Missing	1.13 (1.09 to 1.18)	<0.001
Year trend	0.94 (0.94 to 0.95)	<0.001

BMI, body mass index; CCI, Charlson Comorbidity Index; NDH, non-diabetic hyperglycaemia; T2DM, type 2 diabetes mellitus.

## Discussion

In our cohort, incidence of NDH increased from 0.02% in 2000 to 0.21% in 2015. NDH is more common in males and the proportion with NDH increased with age, up to 75 years. The proportion of individuals diagnosed with NDH increased with BMI. The time taken to convert from NDH to T2DM was further explored which showed that approximately 7% converted to T2DM within a year. The conversion rates were also explored by year from 2000 to 2015, which showed a general trend of a decline in the conversion rate from NDH to T2DM with a peak in the year 2004 and 2011. The risk of conversion from NDH to T2DM was higher in men and those aged 45–54 years, decreasing with age. People with NDH who are overweight, and even more so those who are obese, have a higher risk of developing diabetes. Depression, deprivation and smoking (perhaps as a deprivation proxy) were also modestly associated with T2DM conversion.

Our study has several strengths. It was based on a large, longitudinal and nationally representative data resource. The length of the study period is also useful in capturing changes over time. This study has some limitations. Our diagnosed cases of NDH and T2DM are based on Read codes being used. Although we could have considered other approaches to define NDH and T2DM to avoid false positives, in the context of the UK primary care, coding of T2DM is known to be of very high quality because of the Quality and Outcomes Framework (QOF), which incentive GPs for accurate recording.^14^ Although this change occurred in 2004, quality was already high from 2000 onwards, in anticipation for the scheme and other smaller-scale frameworks. The only potential issue with the QOF was the non-distinction in coding between type 1 and type 2, until explicitly requested in 2006. This may have led to us missing a few cases that exited the database before 2006, if they had type 2 diabetes but were only given a generic diabetes code. In our experience this is very rare, however, and it would not affect our finding that conversion rates for NDH have dropped over time. As previously mentioned, the quality of recording is very high and people associated with a Read code for T2DM, have the condition—there is no provisional coding and GPs are encouraged to add to records only if certain since they know retracting such a diagnosis is very complicated. If someone is suspected of having the condition they will be not be given a Read code, but information will be added in notes (or with a ‘suspected diabetes’ code). Remission is possible of course, although rare, but it is not relevant for this study (where T2DM is the outcome of interest in a time to event analysis).

Regarding NDH coding, the situation is more complicated because of the absence of financial incentives through the QOF, hence practice variability is greater. In addition, the definition of NDH has changed over time, as we explain in the paper, making it difficult to operationalise through biological measurements, which are very often missing.

Estimates from EHRs are sensitive to the code lists and that our findings need to be interpreted with caution,^18^ however, our code list included only a few relevant items and is not sensitive to misclassification. For BMI and cholesterol, we categorise and include a ‘missing’ category, which can be problematic, but allows us to observe the associations with T2DM conversion. Our risk prediction model did not attempt to include and reaffirm all known drivers of diabetes, but we primarily aimed to examine the role of socioeconomic drivers and lifestyle factors, along with depression (potentially actionable and important comorbidity for T2DM,^19^ and a proxy for ‘overall health’. Alcohol intake was not included in the model, since the quality of recording such information in UK primary care is rather poor.^20^ We also decided not to use medication for two reasons: first, we would need to capture and organise everything to a patient (and the relevant volumes), which is a tremendous amount of work, with no clear link to conversion as far as we know; second, and more importantly, including treatment in our model would probably introduce unmeasured confounding, with treatments being associated to conversion when the underlying conditions and the health of the patient are the driving causes.

Our findings suggested the women were at a lower risk of conversion from NDH to T2DM than men. Previous studies have shown that the incidence of diabetes in those diagnosed with pre-diabetes was higher in women.^10^ The difference may be due to different populations studied (two of the three studies were on American Indians and the other was an Australian population). The discrepancies may also be due to the different definition of NDH used.^21^ For example in the Australian study which followed up 5842 participants over 5 years, men categorised as having IFG had a higher incidence of diabetes compared with women (4.0% vs 2.0%), whereas women categorised with IGT had a significantly higher incidence of diabetes than men (4.4% vs 2.9%).^22^

A review^23^ exploring the rates of conversion from IGT to T2DM showed rates ranging from 1.5% per year in Bradford, UK to 7% in Mexicans and Americans. In our study, rates of conversion from NDH to T2DM decreased from 2000to 2015, with peaks in 2004 and 2011. Since studies in primary care data have suggested that the incidence rates of T2DM has stabilised after 2005,^24^ this apparent decrease in conversion rates needs to be interpreted with caution. One possible explanation is changes in the definition of NDH, with different HbA1c ranges used over the study period. Another plausible explanation for the decreasing trends is changes in coding practice, with more people of lower conversion risk being linked with NDH in primary care records. In addition, the peak we observed for 2011 might either be due to the uptake of NHS Health Checks which was introduced in April 2009 and also WHO recommendation in 2011 to use HbA1c for T2DM diagnosis.^25^ A systematic review exploring the trends of pre-diabetes in South Asians, showed that T2DM was rising but the prevalence of IGT was stable or decreasing. They suggested that this might be due to increased testing for T2DM and also studies have found that fasting plasma glucose was more influenced by obesity than 2-hour glucose testing.^26^ It has also been suggested that these decreased trends might be due to a more rapid progression from IGT to T2DM with the IGT state possibly skipping altogether in the disease progression.^27^ Studies have also shown a change of NDH to normoglycaemia after lifestyle and drug-based interventions, which might also be a reason for our findings,^28 29^ as the NICE guidelines have also proposed primary care practitioners to advice patients with NDH on diet and exercise as well as drug interventions with metformin in some cases.^30^ We found a crude rate of conversion of NDH to T2DM to be about 7%, where a previous report using CPRD in which pre-diabetes was defined using Fasting glucose levels showed the progression of IFG to diabetes was 6% per year.^31^

The prevalence of NDH in Health Survey England analyses showed an increase with age, and it increased from 3% in 16–69 age groups to 30.4% in those aged over 80 years.^10^ However, our findings showed the risk of conversion to diabetes from NDH decreased with increasing age and the risk was significantly lower in those aged over 75 years compared with those aged 18–44. Similar associations were shown in The Strong Heart Study which suggested that this might be due to the survival effect in the older adults and the prevalence of obesity being higher in younger adults.^32^ An analysis of six prospective studies which explored the predictors of progression from IGT to non-insulin dependent diabetes mellitus (NIDDM) found inconsistent relationships with age. In the studies with the highest incidence rates of NIDDM, the progression of NIDDM increased with age in participants diagnosed with IGT at a younger age and decreased with age in participants who were diagnosed with IGT at an older age.^33^ There was a negative association in those aged over 85 years and the risk of conversion from NDH to T2DM. This negative association may be due to the fact older population may be less likely to be checked for T2DM in primary care^31^ or the threshold needed to identify NDH in older adults may need to be reconsidered.

We also found the risk of conversion of NDH increased with increase in BMI. Obesity has been linked to increased prevalence of pre-diabetes previously,^34^ however, several other studies exploring the progression of pre-diabetes to T2DM have shown conflicting results with BMI playing a small or non-significant role.^33^

We also showed that current smokers were more likely to convert from NDH to T2DM. In the Health Survey England data, it was shown that the prevalence of pre-diabetes was significantly higher in ex-smokers compared with non-smokers.^10^ Our results also showed a high cholesterol levels were associated with a reduced risk of developing T2DM. Previous studies to our knowledge have not explored the relation of cholesterol with progression of pre-diabetes to diabetes. Our findings also indicated that having a 1–2 Charlson comorbidity score increased the risk of progression to T2DM; however, we were not able to distinguish which comorbidities were linked to progression from NDH to T2DM.

Socioeconomic inequalities exist in healthcare, a fact that has been summarised by Hart’s inverse care law which suggests that those in most need of healthcare are those least likely to receive it.^35^ Our findings that the risk of conversion of NDH to T2DM was higher in those of lower socioeconomic status has not been reported previously, to our knowledge. Although a previous report on NDH by Public Health England using the Health Survey England data showed that there was no significant difference in the prevalence of NDH by quintile of deprivation, the study did not explore the risk of conversion from NDH to T2DM.^10^ Our results align with previous findings which have suggested that IGR/NDH and T2DM are more prevalent in those with low socioeconomic status.^6 7^

## Conclusions

Over the study period, the conversion rate of NDH to T2DM was, on average, 7% within a year. However, there was a large reduction in that rate over time, which should be attributed to changes in coding practices and in the definition of NDH, rather than a reduction in the incidence of T2DM. The key predictors in the progression of NDH to T2DM were age, increased BMI and lower socioeconomic status. It would be interesting to examine the population trends of progression from NDH to T2DM following the introduction of the National Diabetes Prevention Programme, a behavioural intervention programme targeted at people with a high risk of developing T2DM.^9^

## Supplementary Material

Reviewer comments

Author's manuscript
