# Supplementary material for: Epidemiology and determinants of non-diabetic hyperglycaemia and its conversion to type 2 diabetes mellitus, 2000–2015: cohort population study using UK electronic health records
Source: BMJ Open. 2020 Sep 6;10(9):e040201. doi: 10.1136/bmjopen-2020-040201 (PMC7484863; doi:10.1136/bmjopen-2020-040201)
Supplement: Supplementary data [file bmjopen-2020-040201supp001.pdf]

Supplementary

Table 1: Read codes used to diagnose Type 2 Diabetes Mellitus

Medcode	Readcode	Description
506	C100112	Non-insulin dependent diabetes mellitus
758	C10F.00	Type 2 diabetes mellitus
1407	C10FJ00	Insulin treated Type 2 diabetes mellitus
4513	C109.00	Non-insulin dependent diabetes mellitus
5884	C109.11	NIDDM - Non-insulin dependent diabetes mellitus
8403	C109700	Non-insulin dependent diabetes mellitus - poor control
12640	C10FC00	Type 2 diabetes mellitus with nephropathy
12736	C10F500	Type 2 diabetes mellitus with gangrene
17262	C109600	Non-insulin-dependent diabetes mellitus with retinopathy
17859	C109.12	Type 2 diabetes mellitus
18143	C109G11	Type II diabetes mellitus with arthropathy
18209	C109012	Type 2 diabetes mellitus with renal complications
18219	C109.13	Type II diabetes mellitus
18264	C109J12	Insulin treated Type II diabetes mellitus
18278	C109J00	Insulin treated Type 2 diabetes mellitus
18390	C10FM00	Type 2 diabetes mellitus with persistent microalbuminuria
18425	C10FB00	Type 2 diabetes mellitus with polyneuropathy
18496	C10F600	Type 2 diabetes mellitus with retinopathy
18777	C10F000	Type 2 diabetes mellitus with renal complications
22884	C10F.11	Type II diabetes mellitus
24458	C109711	Type II diabetes mellitus - poor control
24693	C109G00	Non-insulin dependent diabetes mellitus with arthropathy
24836	C109C12	Type 2 diabetes mellitus with nephropathy
25041	ZC2CA00	Dietary advice for type II diabetes
25591	C10FQ00	Type 2 diabetes mellitus with exudative maculopathy
25627	C10F700	Type 2 diabetes mellitus - poor control
26054	C10FL00	Type 2 diabetes mellitus with persistent proteinuria
29979	C109900	Non-insulin-dependent diabetes mellitus without complication
32627	C10FN00	Type 2 diabetes mellitus with ketoacidosis
34268	C10F200	Type 2 diabetes mellitus with neurological complications
34450	C10FK00	Hyperosmolar non-ketotic state in type 2 diabetes mellitus
34912	C109400	Non-insulin dependent diabetes mellitus with ulcer
35385	C10FH00	Type 2 diabetes mellitus with neuropathic arthropathy
36633	C109K00	Hyperosmolar non-ketotic state in type 2 diabetes mellitus
36695	C10D.00	Diabetes mellitus autosomal dominant type 2
37648	C109J11	Insulin treated non-insulin dependent diabetes mellitus
37806	C10FF00	Type 2 diabetes mellitus with peripheral angiopathy
40401	C109500	Non-insulin dependent diabetes mellitus with gangrene
42762	C109612	Type 2 diabetes mellitus with retinopathy
43227	C10F311	Type II diabetes mellitus with multiple complications
43785	C109D00	Non-insulin dependent diabetes mellitus with hypoglyca coma
44779	C109E12	Type 2 diabetes mellitus with diabetic cataract
44982	C10FE00	Type 2 diabetes mellitus with diabetic cataract
45467	C109B00	Non-insulin dependent diabetes mellitus with polyneuropathy
45913	C109712	Type 2 diabetes mellitus - poor control
45919	C109212	Type 2 diabetes mellitus with neurological complications
46150	C109512	Type 2 diabetes mellitus with gangrene

Medcode	Readcode	Description
46917	C10FD00	Type 2 diabetes mellitus with hypoglycaemic coma
47315	C10F711	Type II diabetes mellitus - poor control
47321	C10F100	Type 2 diabetes mellitus with ophthalmic complications
47409	C109B11	Type II diabetes mellitus with polyneuropathy
47816	C109H11	Type II diabetes mellitus with neuropathic arthropathy
47954	C10F900	Type 2 diabetes mellitus without complication
48192	C109E11	Type II diabetes mellitus with diabetic cataract
49074	C10F400	Type 2 diabetes mellitus with ulcer
49655	C10F611	Type II diabetes mellitus with retinopathy
49869	C109G12	Type 2 diabetes mellitus with arthropathy
50225	C109011	Type II diabetes mellitus with renal complications
50429	C109100	Non-insulin-dependent diabetes mellitus with ophthalm comps
50527	C10FB11	Type II diabetes mellitus with polyneuropathy
50609	L180600	Pre-existing diabetes mellitus, non-insulin-dependent
50813	C109A11	Type II diabetes mellitus with mononeuropathy
51756	C10FP00	Type 2 diabetes mellitus with ketoacidotic coma
52303	C109000	Non-insulin-dependent diabetes mellitus with renal comps
53392	C10F911	Type II diabetes mellitus without complication
54899	C109F11	Type II diabetes mellitus with peripheral angiopathy
55075	C109411	Type II diabetes mellitus with ulcer
55842	C109200	Non-insulin-dependent diabetes mellitus with neuro comps
56268	C109D11	Type II diabetes mellitus with hypoglycaemic coma
56803	C107400	NIDDM with peripheral circulatory disorder
57278	C10F011	Type II diabetes mellitus with renal complications
58604	C109611	Type II diabetes mellitus with retinopathy
59253	C10FG00	Type 2 diabetes mellitus with arthropathy
59365	C109C00	Non-insulin dependent diabetes mellitus with nephropathy
59725	C109111	Type II diabetes mellitus with ophthalmic complications
60699	C109F12	Type 2 diabetes mellitus with peripheral angiopathy
60796	C10FL11	Type II diabetes mellitus with persistent proteinuria
61071	C109D12	Type 2 diabetes mellitus with hypoglycaemic coma
62107	C109511	Type II diabetes mellitus with gangrene
62146	C109300	Non-insulin-dependent diabetes mellitus with multiple comps
62674	C10FA00	Type 2 diabetes mellitus with mononeuropathy
63690	C10FR00	Type 2 diabetes mellitus with gastroparesis
64571	C109C11	Type II diabetes mellitus with nephropathy
64668	C10FJ11	Insulin treated Type II diabetes mellitus
65267	C10F300	Type 2 diabetes mellitus with multiple complications
65704	C109412	Type 2 diabetes mellitus with ulcer
66965	C109H12	Type 2 diabetes mellitus with neuropathic arthropathy
67905	C109211	Type II diabetes mellitus with neurological complications
69278	C109E00	Non-insulin depend diabetes mellitus with diabetic cataract
70316	C109112	Type 2 diabetes mellitus with ophthalmic complications
72320	C109A00	Non-insulin dependent diabetes mellitus with mononeuropathy
83532	66Ao.00	Diabetes type 2 review
85991	C10FM11	Type II diabetes mellitus with persistent microalbuminuria
91646	C10F411	Type II diabetes mellitus with ulcer
93727	C10FE11	Type II diabetes mellitus with diabetic cataract
95351	C10FA11	Type II diabetes mellitus with mononeuropathy

**Medcode**	**Readcode**	**Description**
98616	C10F211	Type II diabetes mellitus with neurological complications
98723	C10FD11	Type II diabetes mellitus with hypoglycaemic coma
100964	C10F111	Type II diabetes mellitus with ophthalmic complications
101801	66At100	Type II diabetic dietary review
102201	C10FC11	Type II diabetes mellitus with nephropathy
102611	66At111	Type 2 diabetic dietary review
103902	C10FG11	Type II diabetes mellitus with arthropathy
104323	C10F511	Type II diabetes mellitus with gangrene
104639	C10FF11	Type II diabetes mellitus with peripheral angiopathy
105784	C109912	Type 2 diabetes mellitus without complication
106061	C10FP11	Type II diabetes mellitus with ketoacidotic coma
106528	C10FN11	Type II diabetes mellitus with ketoacidosis
107701	C10FK11	Hyperosmolar non-ketotic state in type II diabetes mellitus
108005	C109312	Type 2 diabetes mellitus with multiple complications
109103	C109911	Type II diabetes mellitus without complication
109197	C10FH11	Type II diabetes mellitus with neuropathic arthropathy
109865	C109B12	Type 2 diabetes mellitus with polyneuropathy
111798	C10FQ11	Type II diabetes mellitus with exudative maculopathy

Ravindrarajah R, *et al. BMJ Open* 2020; 10:e040201. doi: 10.1136/bmjopen-2020-040201
