# Supplementary material for: Epidemiology and determinants of non-diabetic hyperglycaemia and its conversion to type 2 diabetes mellitus, 2000–2015: cohort population study using UK electronic health records
Source: BMJ Open. 2020 Sep 6;10(9):e040201. doi: 10.1136/bmjopen-2020-040201 (PMC7484863; doi:10.1136/bmjopen-2020-040201)
Supplement: Author's manuscript [file bmjopen-2020-040201.draft_revisions.pdf]

BMJ Open is committed to open peer review. As part of this commitment we make the peer review history of every article we publish publicly available.

When an article is published we post the peer reviewers' comments and the authors' responses online. We also post the versions of the paper that were used during peer review. These are the versions that the peer review comments apply to.

The versions of the paper that follow are the versions that were submitted during the peer review process. They are not the versions of record or the final published versions. They should not be cited or distributed as the published version of this manuscript.

BMJ Open is an open access journal and the full, final, typeset and author-corrected version of record of the manuscript is available on our site with no access controls, subscription charges or pay-per-view fees (http://bmjopen.bmj.com).

If you have any questions on BMJ Open's open peer review process please email info.bmjopen@bmj.com

BMJ Open

The epidemiology and determinants of non-diabetic hyperglycaemia and its conversion to type 2 diabetes mellitus, 2000-2015: cohort population study using UK electronic health records.

Journal:	*BMJ Open*
Manuscript ID	bmjopen-2020-040201
Article Type:	Original research
Date Submitted by the Author:	12-May-2020

Complete List of Authors:	Ravindrarajah, Rathi; The University of Manchester, Faculty of Biology, Medicine and Health;<br>Reeves, David; University of Manchester, Institute of Population Health; Centre for Biostatistics<br>Howarth, Elizabeth; The University of Manchester, Faculty of Biology, Medicine and Health<br>Meacock, Rachel; Univ Manchester,<br>Soiland-Reyes, Claudia; Salford Royal NHS Foundation Trust; Salford Royal NHS Foundation Trust<br>Cotterill, Sarah; University of Manchester, Centre for Biostatistics<br>Whittaker, William; University of Manchester, Manchester Centre for Health Economics<br>Heller, Simon; Unversity of Sheffield, Academic Unit of Diabetes, Endo and Metab<br>Sutton, Matt; University of Manchester<br>Bower, Peter; University of Manchester, NPCRDC<br>Kontopantelis, Evangelos; The University of Manchester, Faculty of Biology, Medicine and Health

Keywords:	DIABETES & ENDOCRINOLOGY, EPIDEMIOLOGY, PRIMARY CARE

SCHOLARONE™
Manuscripts

I, the Submitting Author has the right to grant and does grant on behalf of all authors of the Work (as defined in the below author licence), an exclusive licence and/or a non-exclusive licence for contributions from authors who are: i) UK Crown employees; ii) where BMJ has agreed a CC-BY licence shall apply, and/or iii) in accordance with the terms applicable for US Federal Government officers or employees acting as part of their official duties; on a worldwide, perpetual, irrevocable, royalty-free basis to BMJ Publishing Group Ltd ("BMJ") its licensees and where the relevant Journal is co-owned by BMJ to the co-owners of the Journal, to publish the Work in this journal and any other BMJ products and to exploit all rights, as set out in our licence.

The Submitting Author accepts and understands that any supply made under these terms is made by BMJ to the Submitting Author unless you are acting as an employee on behalf of your employer or a postgraduate student of an affiliated institution which is paying any applicable article publishing charge ("APC") for Open Access articles. Where the Submitting Author wishes to make the Work available on an Open Access basis (and intends to pay the relevant APC), the terms of reuse of such Open Access shall be governed by a Creative Commons licence – details of these licences and which Creative Commons licence will apply to this Work are set out in our licence referred to above.

Other than as permitted in any relevant BMJ Author's Self Archiving Policies, I confirm this Work has not been accepted for publication elsewhere, is not being considered for publication elsewhere and does not duplicate material already published. I confirm all authors consent to publication of this Work and authorise the granting of this licence.

For peer review only

The epidemiology and determinants of non-diabetic hyperglycaemia and its conversion to type 2 diabetes mellitus, 2000-2015: cohort population study using UK electronic health records.

Rathi Ravindrarajah[a,], David Reeves[a] ,Elizabeth Howarth [a,], Rachel Meacock[a], Soiland-Reyes Claudia[c], Sarah Cotterill[b], William Whittaker[a], Simon Heller[d], Matt Sutton[a], Peter Bower[a], Evangelos Kontopantelis[a]

[a] Division of Population Health, Faculty of Biology, Medicine and Health, University of Manchester
[b] Centre for Biostatistics, School of Health Sciences, University of Manchester
[c] NIHR CLAHRC Greater Manchester, Salford Royal NHS Foundation Trust, Salford, UK
[d] Dept. of Oncology and Metabolism, University of Sheffield

Dr. David Reeves (PhD),Dr. Elizabeth Howarth (PhD), Dr.Rachel Meacock (PhD), Ms. Soiland-Reyes Claudia (MPhil), Dr. Sarah Cotterill (PhD), Dr. William Whittaker (PhD), Pof. Simon Heller (FRCP), Prof. Matt Sutton (PhD), Prof. Peter Bower (PhD), Prof. Evan Kontopantelis (PhD)

Corresponding Author: Rathi Ravindrarajah (PhD),
Research Associate
Division of Population Health
Faculty of Biology, Medicine and Health
University of Manchester
Email id: rathi.ravindrarajah@manchester.ac.uk

For peer review only

Word Count: 3657

Key Words: Non-Diabetic Hyperglycaemia, Prediabetes, Electronic Health Records, Type 2 Diabetes

For peer review only - http://bmjopen.bmj.com/site/about/guidelines.xhtml

Abstract

Objectives

To study the characteristics of UK individuals identified with non-diabetic hyperglycaemia (NDH) and their conversion rates to Type 2 Diabetes Mellitus (T2DM) from 2000 to 2015, using the Clinical Practice Research Datalink (CPRD).

Design

Cohort study

Settings

UK primary Care Practices

Participants

Electronic health records identified 14,272 participants with NDH, from 2000 to 2015

Primary and Secondary Outcome Measures

Baseline characteristics and conversion trends from NDH to T2DM were explored. Cox proportional-hazards models evaluated predictors of conversion.

Results

Crude conversion was 4% within 6 months of NDH diagnosis, 7% annually, 13% within 2 years, 17% within 3 years and 23% within 5 years. However, 1-year conversion fell from 8% in 2000 to 4% in 2014. Individuals aged 45-54 were at the highest risk of developing T2DM (HR= 1.20; 95% CI: 1.15, 1.25 – compared to those aged 18-44), and the risk reduced with older age. A BMI above 30 kg/m^2 was strongly associated with conversion (HR=2.02; 95% CI: 1.92, 2.13 – compared to those with a normal BMI). Depression (HR=1.10; 95% CI: 1.07, 1.13), smoking (HR=1.07; 95% CI: 1.03, 1.11 – compared to non-smokers) or residing in the most deprived areas (HR=1.17; 95% CI: 1.11, 1.24 – compared to residents of the most affluent areas) was modestly associated with conversion.

Conclusion

Although the rate of conversion from NDH to T2DM fell between 2010 and 2015, this is likely due to changes over time in the cut-off points for defining NDH, and more people of lower diabetes risk being diagnosed with NDH over time. People aged 45-54, smokers, depressed, with high BMI, and more deprived are at increased risk of conversion to T2DM.

Funding

This manuscript is independent research funded by the National Institute for Health Research (Health Services and Delivery Research, 16/48/07 – Evaluating the NHS Diabetes Prevention Programme (NHS DPP): the DIPLOMA research programme (Diabetes Prevention – Long Term Multimethod Assessment)).

For peer review only

For peer review only - http://bmjopen.bmj.com/site/about/guidelines.xhtml

Strengths and limitations of the Study

- Data was based on a large, anonymised, longitudinal and nationally representative sample of general practices
- The length of the study period (2000 to 2015) was useful in capturing changes over time
- Cases of NDH and T2DM were identified using Read codes, and the quality of recording may have been problematic for the former in earlier years
- Our NDH code list included a few relevant items and is not sensitive to misclassification

For peer review only

For peer review only - http://bmjopen.bmj.com/site/about/guidelines.xhtml

Introduction

The proportion of the population with type-2 diabetes mellitus (T2DM) has been rising globally and is an important contributor to mortality, morbidity and health care costs. It has been estimated that 415m people live with diabetes across the globe and 193m people have undiagnosed diabetes [1]. It has been suggested that currently there are 5 million people in England who are at risk of developing T2DM [2]. T2DM is characterized by pancreatic dysfunction causing insulin resistance. There are other key pathophysiological processes which increase the risk of T2DM, which involves organs including pancreas, liver, skeletal muscle, kidneys, brain, small intestine and adipose tissue[3]. Lifestyle factors such as excess weight and physical inactivity are known to increase the risk of developing T2DM.

Non-diabetic hyperglycaemia (NDH also known as pre-diabetes or impaired glucose regulation), refers to levels of blood glucose that are increased from the normal range but not yet high enough to be in the diabetic range. Previous research has shown that individuals diagnosed with NDH are at a higher risk of developing T2DM [4]. The NHS RightCare diabetes pathway defines NDH as having an HbA1c measurement in the 42-47 mmol/mol range (6.0-6.4%), or fasting plasma glucose in the 5.5-6.9 mmol/mol range [5]. Previous analyses using Health Survey England data have shown discrepancies in the prevalence of NDH in the UK. While one study suggested that the average NDH prevalence was 11% in adults aged 16+ in England, in the period between 2009 and 2013 [6], the other suggested a sharp rise in the prevalence of NDH from 11.6% in 2003 to 35.3% in 2011 in all adults [7]. The use of different cut-points for HbA1C used to define NDH has been suggested as the cause of this discrepancy; one study used the NICE and Diabetes UK cut-points (HbA1C: 42-47 mmol/mol) whereas the other used the American Diabetes Association cut-points (HbA1C: 39-47 mmol/mol). Delaying or preventing T2DM has become an international priority due to the burden that the condition places on both patients and health services [8]. NHS England, Public Health England and Diabetes UK have implemented a programme to identify those at high risk of developing T2DM and offer them an evidence-based behavioural intervention (NHS Diabetes Prevention Programme: NHS DPP) to people identified as having NDH in an attempt to reduce the incidence of T2DM and the complications related to it [9].

This paper explores two aspects of the epidemiology of people diagnosed with NDH in UK primary care. First, we aimed to estimate the prevalence of NDH and to explore the characteristics of patients with NDH in a population cohort of adults from 2000 until 2015. We chose this study period both to ensure high data quality and to avoid introducing bias into our analysis from any potential effects from

For peer review only

the National Diabetes Prevention Programme [10]. Second, we evaluated the conversion rates of NDH to T2DM over time, and whether conversion rates differ by age, sex, BMI levels, depression, multi-morbidity and area level deprivation.

Methods

Data Source

Patient level data was obtained from the Clinical Practice Research Datalink (CPRD), one of the largest active primary care databases of electronic health records (EHR) in the UK [11]. This dataset captures approximately 7% of the total UK population. The database holds anonymised data which contains information on clinical signs, diagnoses, tests and procedures [11]. Approximately 60% of all UK CPRD practices participate in the CPRD linkage scheme, which provides additional patient-level information. For this work, we obtained patient-level deprivation through the Office of National Statistics (ONS) linkage, in the form of the 2010 Index of Multiple Deprivation (IMD) [12].

Study Participants

Practices taking part in the CPRD are checked for eligibility in each year using a CPRD assessment algorithm, and evaluated to be of research standard or not. Patients were regarded as eligible if they had been registered with a practice for a full year, were aged 18 years and over and had a code for NDH between 1st April 2000 and 31st March 2016. At least one relevant Read code was considered adequate to flag a patient. Codes were identified using a strategy that involved searching for relevant terms through an algorithm, with the returned list being reviewed and finalised by members of the research team, as described elsewhere [13 14]. Read codes which were actively used by GPs to identify NDH were included in the study: 44v2.00 (Glucose Tolerance Test impaired), C11y200 (Impaired glucose tolerance), C11y300 (Impaired fasting glycaemia), C11y500 (Pre-diabetes), C317.00 (Non-diabetic Hyperglycaemia), R102.00 ([D] Glucose Tolerance Test abnormal), R102.11 ([D] Prediabetes), R102.12 ([D] Impaired glucose tolerance test), R10D000 ([D] Impaired fasting glycaemia), R10D011 ([D] Impaired fasting glucose), R10E.00 ([D] Impaired glucose tolerance. Eligible patients were followed up until censored at the earliest of any of the following events: diagnosed with T2DM (the outcome event), transferred out of practice (any cause), last collection date for the practice, end date of the study (31st March 2016) or death. To report prevalence, we also included cases that were diagnosed with NDH at any point prior to 1st April 2000, who met all other inclusion criteria.

Study measures

We calculated the prevalence of NDH in each year between 2000 and 2015, and conversion to T2DM was also determined. People with at least one relevant Read code of T2DM following the NDH

For peer review only - http://bmjopen.bmj.com/site/about/guidelines.xhtml

diagnosis (the index date), were considered to have progressed to T2DM during the study period (Supplement Table 1 provides a list of read codes used to diagnose T2DM). Patients with a previous record of Type-1 Diabetes were excluded.

We extracted information on the following covariates which have previously been reported [10] to be relevant to NDH and T2DM; age, gender, BMI, total serum cholesterol, smoking status, socio economic status and depression. Age was grouped into the following bands: 18-34, 35-44, 45-54, 55-64, 65-74, 75-84, and 85 years or over. The latest available measurement before the NDH diagnosis date, up until the previous 12 months, was used to define baseline total cholesterol and BMI. If such a value was not available, the measurement was set to missing. BMI values were classified into the following categories: underweight (<18.5 kg/m^2), normal weight (18.5-24.9 kg/m^2), overweight (25.0-29.9 kg/m^2) and obese (>=30 kg/m^2). Total serum cholesterol in mmol/l was categorised into: under 3.0, [3.0, 4.0), [4.0, 5.0), [5.0, 6.0) and 6.0 or over. We also quantified the multi-morbidity burden, using the Charlson Comorbidity Index (CCI), which is a widely used measure which assigns different weights to different conditions and includes: any malignancy, cerebrovascular disease, chronic pulmonary disease, congestive cardiac disease, dementia, HIV/AIDS, hemiplegia, lymphoproliferative disorders, metastatic solid tumour, mild liver disease, moderate and severe liver disease (CCI also includes diabetes with complications, which we necessarily excluded)[15 16]. This modified CCI was calculated using the list of validated diagnostic primary care Read codes used by Khan et al [15]. Participants were classified as having a condition if the condition was present at diagnosis of NDH or 12 months prior to diagnosis of NDH. CCI takes integer values and was categorised as: 0, 1 to 2, 3 to 4 and greater than 4. Depression was evaluated using medical codes and therapy codes which were obtained from the code lists derived from the CPRD provided on a Cambridge University repository [17]. Participants were considered to have depression at the index date (the date of NDH diagnosis) if they were recorded as depressed either by a code or if they were on relevant medication in the last 12 months. Smoking status was determined from information in the patients' record and categorised as "smoker", "ex-smoker" or "never smoked". The Index of Multiple Deprivation (IMD) was used to classify deprivation and the IMD scores were divided into quintiles.

Conversion of NDH to Type 2 Diabetes Mellitus

The time of conversion of NDH to T2DM was defined as the time from the index date (diagnosis of NDH) to the date they were diagnosed as having T2DM. This time was then categorised into progression time of: 1 month; 3 months, 6 months, 12 months, 2 years, 3 years, 4 years, and 5 years. Those who had a conversion time of over 5 years were excluded from analysis. In addition, patients who did not convert to T2DM, left the study or died within this study period were categorised into a

For peer review only

single category as “Not converted/left/died”. A small number of participants were diagnosed as having T2DM on, or ever before, the index date, and were excluded from further analyses (See Figure 1).

Statistical Analysis

The characteristics of people identified with NDH are presented descriptively. Conversion rates of NDH to T2DM, in the progression time categories were plotted over time. Annual bins were defined as financial years, for example 1st April 2000 to 31st March 2001 was labelled as 2000. The associations between covariates and conversion from NDH to T2DM were estimated in a time to event analysis. A Cox proportional hazards model was employed to estimate adjusted hazard ratios (HRs) of the associations between conversion and the following covariates: gender, age groups, BMI categories, total cholesterol levels, depression, year, patient-level deprivation scores and CCI categories. Proportionality of hazards was tested using Schoenfeld residuals.

Results

Over the study period, a total of 148,363 participants were identified with NDH. The prevalence and incidence of NDH for each financial year is shown in Table 1. Prevalence increased from 0.07% in 2000 to 1.85% in 2015. Incidence of NDH increased from 0.02% in 2000 to 0.21% in 2015. Table 2 and Figure 2 show the cumulative frequency of conversion from NDH to T2DM, by year, from 1 April 2000 to 31 March 2016. Frequency of conversion within one financial year peaks in 2003 and then follows a decreasing trend. Amongst this general trend of declining conversion, there was a peak in the year 2011, with a further exploration of the data (results not shown) suggesting that patients had somewhat higher BMIs in this year, although that does not fully explain the rise.

After all exclusion criteria were applied (see Figure 1), our final NDH population was 141,272 people, with a mean follow-up period of 5 years since the index date.

Table 3 displays the baseline characteristics of the cohort. Covariates are treated as categorical variables in our analysis, and so reported here as numbers and percentages. The mean age of the cohort was 63.2 (SD=13.4) years, and 53% were male. The prevalence of NDH was highest in those aged 65-74 years (39,178/141,272; 27.7%). The proportion of NDH was higher in older females (3728/67,369, 5.5%), compared to older males (2162/73,903; 2.9%) aged 85 years and more. The most common BMI category in our cohort was obese, with 32% of females with a measurement of BMI equal to or above 30 kg/m^2. Results showed that 19% of the NDH cohort had depression when they were diagnosed with NDH. The vast majority of the NDH population (85%) had a Charlson comorbidity score of zero at the index date, indicating absence of major comorbidities.

For peer review only - http://bmjopen.bmj.com/site/about/guidelines.xhtml

Table 4 shows the number of patients who converted from NDH to T2DM. Over the whole of the study period, the conversion rates were: 1.6% within 1 month, 3% within 3 months, 4.2% within 6 months, 7% within a year, 12.8% within 24 months, 17.2% within 3 years, 20.4% within 4 years and 22.8% over 5 years. The majority (77.2%, n=104,030) did not convert, but the length of time each was followed up varied depending on the time they were diagnosed with NDH.

Table 5 shows the results from the Cox proportional hazard models, which explored time to conversion from NDH to T2DM, with failure being the diagnosis of T2DM. Residuals were linear over time, indicating that proportionality generally stood. The rate of conversion was highest for the 45-54 age-group with HR=1.20 (95% CI 1.15 to 1.25), compared to those aged 18-44, and the risk steadily decreased with increasing age to a HR of 0.65 (95% CI 0.60 to 0.71) for people aged 85 or over. Cholesterol categories did not appear to be strongly associated with conversion to T2DM. People with high BMI had a much higher risk of conversion to T2DM, with those classed overweight (BMI 25-30) having a HR of 1.40 (95% CI: 1.33 to 1.48), and those classed obese (BMI>=30) having a HR of 2.0 (95% CI: 1.9, 2.1), compared to individuals with a normal BMI (18.5 to 25). Compared to non- smokers, current smokers had a slightly increased risk of converting to T2DM with a HR of 1.07 (95% CI of 1.03 to 1.11). Those who had a CCI score of 1 to 2 had a slightly higher risk of conversion to T2DM with a HR of 1.1 (95% CI: 1.08 to 1.15) but there was no increased risk among those with higher CCI scores. Having depression at baseline slightly increased the risk of conversion (HR=1.10, 95% CI 1.07, 1.13). The risk of conversion to T2DM increased with patient level deprivation as measured by the 2010 IMD, suggesting that those living in more deprived areas are at an increased risk of conversion from NDH to T2DM. Patients living in the least affluent quintile had an HR of 1.17 (95% CI 1.11 to 1.24), compared to patients living in the most affluent quintile.

Discussion

In our cohort, incidence of NDH increased from 0.02% in 2000 to 0.21% in 2015. NDH is more common in males and the proportion with NDH increased with age, up to 75 years. The proportion of individuals diagnosed with NDH increased with BMI. The time taken to convert from NDH to T2DM was further explored which showed that approximately 7% converted to T2DM within a year. The conversion rates were also explored by year from 2000 till 2015, which showed a general trend of a decline in the conversion rate from NDH to T2DM with a peak in the year 2004 and 2011. The risk of conversion from NDH to T2DM was higher in men and those aged 45 to 54 years, decreasing with age. People with NDH who are overweight, and even more so those who are obese, have a higher risk of developing diabetes. Depression, deprivation and smoking (perhaps as a deprivation proxy) were also modestly associated with T2DM conversion.

For peer review only - http://bmjopen.bmj.com/site/about/guidelines.xhtml

For peer review only

Our study has several strengths. It was based on a large, longitudinal and nationally representative data resource. The length of the study period is also useful in capturing changes over time. This study has some limitations. Our diagnosed cases of NDH and T2DM are based on Read codes being used. For BMI and cholesterol, we categorise and include a "missing" category, which can be problematic, but allows us to observe the associations with T2DM conversion. Estimates from EHRs are sensitive to the code lists and that our findings need to be interpreted with caution [18], however, our code list included only a few relevant items and is not sensitive to misclassification. Our risk prediction model did not attempt to include and reaffirm all known drivers of diabetes, but we primarily aimed to examine the role of socio-economic drivers and lifestyle factors, along with depression (potentially actionable and important comorbidity for T2DM [19]), and a proxy for "overall health". Alcohol intake was not included in the model, since the quality of recording such information in UK primary care is rather poor [20]. We also decided not to use medication for two reasons: first, we would need to capture and organise everything to a patient (and the relevant volumes), which is a tremendous amount of work, with no clear link to conversion as far as we know; secondly, and more importantly, including treatment in our model would probably introduce unmeasured confounding, with treatments being associated to conversion when the underlying conditions and the health of the patient are the driving causes.

Our findings suggested the women were at a lower risk of conversion from NDH to T2DM than men. Previous studies have shown that the incidence of diabetes in those diagnosed with prediabetes was higher in women [10]. The difference may be due to different populations studied (two of the three studies were on American Indians and the other was an Australian population). The discrepancies may also be due to the different definition of NDH used [21]. For example in the Australian study which followed up 5,842 participants over 5 years, men categorised as having impaired fasting glucose had a higher incidence of diabetes compared to women (4.0% vs 2.0%), whereas women categorised with impaired glucose tolerance (IGT) had a significantly higher incidence of diabetes than men (4.4% vs. 2.9%) [22].

A review [23] exploring the rates of conversion from IGT to T2DM showed rates ranging from 1.5% per year in Bradford, UK to 7% in Mexicans and Americans. In our study, rates of conversion from NDH to T2DM decreased from 2000 till 2015, with peaks in 2004 and 2011. Since studies in primary care data have suggested that the incidence rates of T2DM has stabilised after 2005, [24] this apparent decrease in conversion rates needs to be interpreted with caution. One possible explanation is changes in the

For peer review only - http://bmjopen.bmj.com/site/about/guidelines.xhtml

definition of NDH, with different HbA1c ranges used over the study period. Another plausible explanation for the decreasing trends is changes in coding practice, with more people of lower conversion risk being linked with NDH in primary care records. In addition, the peak we observed for 2011 might either be due to the uptake of NHS Health Checks which was introduced in April 2009 and also the WHO recommendation in 2011 to use HbA1c for T2DM diagnosis [25]. A systematic review exploring the trends of prediabetes in South Asians, showed that T2DM was rising but the prevalence of IGT was stable or decreasing. They suggested that this might be due to increased testing for T2DM and also studies have found that fasting plasma glucose was more influenced by obesity than 2-hour glucose testing [26]. It has also been suggested that these decreased trends might be due to a more rapid progression from IGT to T2DM with the IGT state possibly skipping altogether in the disease progression [27]. Studies have also shown a change of NDH to normoglycaemia after lifestyle and drug-based interventions, which might also be a reason for our findings [28 29], as the NICE guidelines have also proposed primary care practitioners to advice patients with NDH on diet and exercise as well as drug interventions with metformin in some cases [30]. We found a crude rate of conversion of NDH to T2DM to be about 7%, where a previous report using CPRD in which prediabetes was defined using Fasting glucose levels showed the progression of IFG (Impaired fasting glucose) to diabetes was 6% per year [31].

The prevalence of NDH in Health Survey England analyses showed an increase with age, and it increased from 3% in 16-69 age groups to 30.4% in those aged over 80 years [10]. However, our findings showed the risk of conversion to diabetes from NDH decreased with increasing age and the risk was significantly lower in those aged over 75 years compared to those aged 18-44. Similar associations were shown in The Strong Heart Study which suggested that this might be due to the survival effect in the older adults and the prevalence of obesity being higher in younger adults [32]. An analysis of six prospective studies which explored the predictors of progression from Impaired Glucose Tolerance (IGT) to Non-Insulin Dependent Diabetes Mellitus (NIDDM) found inconsistent relationships with age. In the studies with the highest incidence rates of NIDDM, the progression of NIDDM increased with age in participants diagnosed with IGT at a younger age and decreased with age in participants who were diagnosed with IGT at an older age [33].There was a negative association in those aged over 85 years and the risk of conversion from NDH to T2DM. This negative association may be due to the fact older population may be less likely to be checked for T2DM in primary care [31] or the threshold needed to identify NDH in older adults may need to be reconsidered.

For peer review only - http://bmjopen.bmj.com/site/about/guidelines.xhtml

We also found the risk of conversion of NDH increased with increase in BMI. Obesity has been linked to increased prevalence of prediabetes previously [34], however several other studies exploring the progression of prediabetes to T2DM have shown conflicting results with BMI playing a small or non-significant role [33].

We also showed that current smokers were more likely to convert from NDH to T2DM. In the Health Survey England data it was shown that the prevalence of prediabetes was significantly higher in ex-smokers compared to non-smokers [10]. Our results also showed a high cholesterol levels were associated with a reduced risk of developing T2DM. Previous studies to our knowledge have not explored the relation of cholesterol with progression of prediabetes to diabetes. Our findings also indicated that having a 1-2 Charlson comorbidity score increased the risk of progression to T2DM; however, we were not able to distinguish which co-morbidities were linked to progression from NDH to T2DM.

Socioeconomic inequalities exist in health care, a fact that has been summarised by Hart's inverse care law which suggests that those in most need of health care are those least likely to receive it [35]. Our findings that the risk of conversion of NDH to T2DM was higher in those of lower socioeconomic status has not been reported previously, to our knowledge. Although a previous report on NDH by Public Health England using the Health Survey England data showed that there was no significant difference in the prevalence of NDH by quintile of deprivation, the study did not explore the risk of conversion from NDH to T2DM [10]. Our results align with previous findings which have suggested that impaired glucose regulation (IGR/NDH) and T2DM are more prevalent in those with low socioeconomic status [6 7].

Conclusions

Over the study period, the conversion rate of NDH to T2DM was, on average, 7% within a year. However, there was a large reduction in that rate over time, which should be attributed to changes in coding practices and in the definition of NDH, rather than a reduction in the incidence of T2DM. The key predictors in the progression of NDH to T2DM were age, increased BMI and lower socioeconomic status. It would be interesting to examine the population trends of progression from NDH to T2DM following the introduction of the National Diabetes Prevention Programme, a behavioural intervention programme targeted at people with a high risk of developing T2DM [9].

For peer review only - http://bmjopen.bmj.com/site/about/guidelines.xhtml

Table 1: Prevalence and Incidence of NDH

	Prevalence			Incidence		
Year	Numerator	Denominator	%	Numerator	Denominator	%
2000	2809	3,784,862	0.07	750	3,782,803	0.02
2001	4065	3,825,769	0.11	1256	3,822,960	0.03
2002	6627	3,868,575	0.17	2562	3,864,510	0.07
2003	10,790	3,905,077	0.28	4163	3,898,450	0.11
2004	16,687	3,957,556	0.42	5897	3,946,766	0.15
2005	23,989	3,996,114	0.60	7302	3,979,427	0.18
2006	29,805	4,029,795	0.74	5816	4,005,806	0.15
2007	35,730	4,074,123	0.88	5925	4,044,318	0.15
2008	41,930	4,130,943	1.02	6200	4,095,213	0.15
2009	48,116	4,191,018	1.15	6186	4,149,088	0.15
2010	52,891	4,245,410	1.25	4775	4,197,294	0.11
2011	57,556	4,283,200	1.34	4665	4,230,309	0.11
2012	61,787	4,335,322	1.43	4231	4,277,766	0.10
2013	68,376	4,383,749	1.56	6589	4,321,962	0.15
2014	74,423	4,446,718	1.67	6047	4,378,342	0.14
2015	83,652	4,528,613	1.85	9229	4,454,190	0.21

Note: Year 2000 defined as 01st April 2000 till 31st March 2001 and other years defined similarly

For peer review only

For peer review only - http://bmjopen.bmj.com/site/about/guidelines.xhtml

Table 2: Cumulative frequency of conversion from NDH to T2DM from 2000 to 2015

	Within 1 month				Within 3 months				Within 6 months				Within 1 year			
**Year**	**N remaining unconverted**	**N converted**	**N censored**	**Cum % converted**	**N remaining unconverted**	**N converted**	**N censored**	**Cum % converted**	**N remaining unconverted**	**N converted**	**N censored**	**Cum % converted**	**N remaining unconverted**	**N converted**	**N censored**	**Cum % converted**
**2000**	887	19	1	2.10	870	13	4	3.53	854	15	1	5.20	818	25	11	7.99
**2001**	1460	35	0	2.34	1433	26	1	4.08	1397	29	7	6.03	1320	58	19	9.96
**2002**	2922	72	2	2.40	2863	55	4	4.24	2803	47	13	5.82	2650	126	27	10.07
**2003**	4793	115	5	2.34	4655	125	13	4.89	4538	85	32	6.63	4276	183	79	10.43
**2004**	7076	184	6	2.53	6907	151	18	4.62	6698	160	49	6.83	6370	241	87	10.21
**2005**	8832	185	7	2.05	8660	152	20	3.74	8479	132	49	5.21	8007	335	137	8.99
**2006**	8561	193	4	2.20	8389	149	23	3.91	8194	140	55	5.52	7743	319	132	9.23
**2007**	9240	192	14	2.03	9073	144	23	3.56	8912	130	31	4.95	8472	317	123	8.35
**2008**	10243	179	10	1.72	10046	172	25	3.37	9871	114	61	4.47	9391	370	110	8.07
**2009**	10923	191	8	1.72	10721	185	17	3.38	10553	123	45	4.49	10100	319	134	7.40
**2010**	9991	189	4	1.86	9828	146	17	3.29	9686	107	35	4.35	9279	291	116	7.24
**2011**	9973	163	6	1.61	9792	161	20	3.20	9628	126	38	4.45	9181	309	138	7.53
**2012**	10057	162	5	1.58	9912	130	15	2.86	9743	131	38	4.14	9366	274	103	6.85
**2013**	12267	131	17	1.06	12130	110	27	1.94	11963	115	52	2.88	11537	264	162	5.03
**2014**	11318	85	14	0.74	11214	71	33	1.37	11061	92	61	2.18	10717	209	135	4.04
**2015**	12832	81	1080	0.60	10111	85	2636	1.34	6716	72	3323	2.18				

For peer review only

For peer review only - http://bmjopen.bmj.com/site/about/guidelines.xhtml

Table 2 contd: Cumulative frequency of conversion from NDH to T2DM from 2000 to 2015

	Within 2 years				Within 3 years				Within 4 years				Within 5 years			
**Year**	**N remaining unconverted**	**N converted**	**N censored**	**Cum % converted**	**N remaining unconverted**	**N converted**	**N censored**	**Cum % converted**	**N remaining unconverted**	**N converted**	**N censored**	**Cum % converted**	**N remaining unconverted**	**N converted**	**N censored**	**Cum % converted**
**2000**	734	62	22	15.06	634	68	32	23.10	545	57	32	30.20	456	60	29	38.09
**2001**	1160	103	57	17.14	971	135	54	27.01	827	94	50	34.26	694	76	57	40.52
**2002**	2283	256	111	18.95	1973	210	100	26.57	1674	198	101	34.13	1377	191	106	41.89
**2003**	3647	437	192	19.80	3105	359	183	27.89	2672	272	161	34.38	2305	228	139	40.13
**2004**	5490	590	290	18.72	4726	471	293	25.88	4086	384	256	32.07	3533	325	228	37.63
**2005**	6939	711	357	17.25	6025	577	337	24.30	5275	459	291	30.21	4650	406	219	35.70
**2006**	6741	700	302	17.60	5841	638	262	25.55	5076	467	298	31.66	4468	341	267	36.37
**2007**	7328	829	315	17.49	6385	643	300	24.88	5612	484	289	30.71	4959	379	274	35.50
**2008**	8176	836	379	16.42	7247	602	327	22.70	6473	474	300	27.86	5763	421	289	32.66
**2009**	9059	708	333	14.00	8049	621	389	20.02	7229	500	320	25.09	6597	344	288	28.73
**2010**	8324	616	339	13.51	7427	587	310	19.73	6712	440	275	24.57	6186	306	220	28.07
**2011**	8091	773	317	15.46	7303	473	315	20.50	6703	342	258	24.29	0	137	6566	27.32
**2012**	8467	537	362	12.30	7769	366	332	16.17								
**2013**	10625	487	425	9.12												

For peer review only - http://bmjopen.bmj.com/site/about/guidelines.xhtml

Table 3: Characteristics of the cohort

	All	Males	Females
**N**	141,272	73,903 (52.3)	67,369 (47.7)
**Age (years)**	63.2±13.4	62.8±12.4	63.6±14.5
**Age group**	Count (%)		
**18-44**	12,896 (9.1)	5619 (7.6)	7277 (10.8)
**45-54**	22,717 (16.1)	12,934 (17.5)	9783 (14.5)
**55-64**	36,790 (26.0)	21,127 (28.6)	15,663 (23.3)
**65-74**	39,178 (27.7)	21,042 (28.5)	18,136 (26.9)
**75-84**	23,801 (16.9)	11,019 (14.9)	12,782 (19.0)
**>=85**	5890 (4.2)	2162 (2.9)	3728 (5.5)
**Smoking Status**	Count (%)		
**Current**	21,088 (14.9)	11,352 (15.4)	9736 (14.5)
**Ex**	46,301 (32.8)	27,979 (37.9)	18,322 (27.2)
**Never**	27,834 (19.7)	12,046 (16.3)	15,788 (23.4)
**Missing**	46,049 (32.6)	22,526 (30.5)	23,523 (34.9)
**BMI Categories (kg/m²)**	Count (%)		
**<18.5**	628 (0.4)	153 (0.2)	475 (0.7)
**18.5-25**	11,553 (8.2)	5504 (7.5)	6049 (9.0)
**25-30**	27,523 (19.5)	16,686 (22.6)	10,837 (16.1)
**>=30**	42,456 (30.1)	21,189 (28.7)	21,267 (31.6)
**Missing**	59,112 (41.8)	30,371 (41.1)	28,741 (42.7)
**Cholesterol (%)**	Count (%)		
**<3**	1538 (1.1)	1203 (1.6)	336 (0.5)
**3 to 4**	12,668 (9.0)	8814 (11.9)	3859 (5.7)
**4 to 5**	29,204 (20.7)	17,170 (23.2)	12,041 (17.9)
**5 to 6**	28,554 (20.2)	14,889 (20.1)	13,670 (20.3)
**>=6**	22,818 (16.2)	9844 (13.3)	12,979 (19.3)
**Missing**	46,490 (32.9)	22,002 (29.8)	24,513 (36.4)
**Depression**	26,064 (18.5)	9724 (13.2)	16,340 (24.3)
**CCI Score**	Count (%)		
**None**	120,158 (85.1)	63,571 (86.0)	56,587 (84.0)
**1 to 2**	20,912 (14.8)	10,215 (13.8)	10,697 (15.9)
**3 to 4**	142 (0.1)	85 (0.1)	57 (0.1)
**>4**	60 (0.04)	32 (0.04)	28 (0.04)
**Patient level deprivation Index (2010 IMD score)**		Count (%)	
**Quintile 1(Most Affluent)**	12,854 (9.1)	7034 (9.5)	5820 (8.6)
**Quintile 2**	13,617 (9.6)	7368 (10.0)	6249 (9.3)
**Quintile 3**	12,882 (9.1)	6692 (9.1)	6190 (9.2)
**Quintile 4**	12,816 (9.1)	6514 (8.8)	6302 (9.4)
**Quintile 5(Least Affluent)**	9866 (7.0)	4780 (6.5)	5086 (7.6)
**Missing**	79,237 (56.1)	41,515 (56.2)	37,722 (56.0)

For peer review only - http://bmjopen.bmj.com/site/about/guidelines.xhtml

Table 4: Conversion from at risk of diabetes (NDH) to T2DM

Time taken to convert from at risk to T2Diabetes	Numerator (total number diagnosed with T2D)	Denominator (total number with NDH)	%	% Change
Within 1 month	2,176	134,734	1.62	
Within 3months	4,051	134,734	3.01	1.39
Within 6months	5,669	134,734	4.21	1.20
Within 1 year	9,369	134,734	6.95	2.75
Within 2 years	17,216	134,734	12.78	5.82
Within 3 years	23,168	134,734	17.20	4.42
Within 4 years	27,490	134,734	20.40	3.21
Within 5 years	30,704	134,734	22.79	2.39

For peer review only

For peer review only - http://bmjopen.bmj.com/site/about/guidelines.xhtml

Table 5: Cox proportional hazard models exploring time to conversion from NDH to T2DM for patients by baseline characteristics

	HR (95% CI)	p value
**Males**	Ref	
**Females**	0.97 (0.95 to 0.99)	0.009
**Age Group (Years)**		
**18-44**	Ref	
**45-54**	1.20 (1.15 to 1.25)	<0.001
**55-64**	1.10 (1.06 to 1.14)	<0.001
**65-74**	1.03 (0.99 to 1.07)	0.13
**75-84**	0.86 (0.82 to 0.90)	<0.001
**>=85**	0.65 (0.60 to 0.71)	<0.001
**Cholesterol categories (%)**		
**<3**	1.04 (0.95 to 1.16)	0.391
**3 to 4**	1.03 (0.99 to 1.07)	0.165
**4 to 5**	Ref	
**5 to 6**	0.94 (0.92 to 0.98)	0.001
**>=6**	0.92 (0.89 to 0.95)	<0.001
**Missing**	0.91 (0.89 to 0.94)	<0.001
**Smoking Status**		
**Non smoker**	Ref	
**Current Smoker**	1.07 (1.03 to 1.11)	<0.001
**Ex- smoker**	0.98 (0.96 to 1.01)	0.312
**missing**	0.98 (0.95 to 1.02)	0.338
**BMI Categories(kg/m^2)**		
**<18.5**	0.59 (0.44 to 0.78)	<0.001
**18.5-25**	Ref	
**25-30**	1.40 (1.33 to 1.48)	<0.001
**>=30**	2.02 (1.92 to 2.13)	<0.001
**Missing**	1.44 (1.37 to 1.52)	<0.001
**Depression**	1.10 (1.07 to 1.13)	<0.001
**CCI Score**		
**None**	Ref	
**1 to 2**	1.11 (1.08 to 1.15)	<0.001
**3 to 4**	0.98 (0.68 to 1.43)	0.934
**>4**	1.67 (0.99 to 2.81)	0.057
**Patient level Deprivation Index**		
**Quintile 1(Most Affluent)**	Ref	
**Quintile 2**	1.08 (1.03 to 1.13)	0.002
**Quintile 3**	1.03 (0.98 to 1.08)	0.237
**Quintile 4**	1.12 (1.07 to 1.18)	<0.001
**Quintile 5(Least Affluent)**	1.17 (1.11 to 1.24)	<0.001
**Missing**	1.13 (1.09 to 1.18)	<0.001
**Year trend**	0.94 (0.94 to 0.95)	<0.001

For peer review only - http://bmjopen.bmj.com/site/about/guidelines.xhtml

Figure 1: Flow diagram of patient inclusions

Figure 2: Cumulative conversion of NDH to T2DM diabetes from 2000 till 2015

For peer review only

References

1. Worldwide trends in diabetes since 1980: a pooled analysis of 751 population-based studies with 4.4 million participants. *The Lancet* 2016;387(10027):1513-30. doi: 10.1016/S0140-6736(16)00618-8
2. Hudson H. The NHS Heath Check screening and non-diabetic hyperglycaemia. *Practice Nursing* 2016;27(10):473-80. doi: 10.12968/pnur.2016.27.10.473
3. Defronzo RA. Banting Lecture. From the triumvirate to the ominous octet: a new paradigm for the treatment of type 2 diabetes mellitus. *Diabetes* 2009;58(4):773-95. doi: 10.2337/db09-9028
4. Federation ID. IDF Diabetes Atlas. 8th Edition ed: Brussels, Belgium, 2017.
5. NHS. NHS RightCare Pathway: Diabetes. In: England N, ed., 2018:1-21.
6. Moody A, Cowley G, Ng Fat L, et al. Social inequalities in prevalence of diagnosed and undiagnosed diabetes and impaired glucose regulation in participants in the Health Surveys for England series. *BMJ open* 2016;6(2) doi: 10.1136/bmjopen-2015-010155
7. Mainous AG, 3rd, Tanner RJ, Baker R, et al. Prevalence of prediabetes in England from 2003 to 2011: population-based, cross-sectional study. *BMJ open* 2014;4(6):e005002. doi: 10.1136/bmjopen-2014-005002 [published Online First: 2014/06/11]
8. Barry E, Roberts S, Oke J, et al. Efficacy and effectiveness of screen and treat policies in prevention of type 2 diabetes: systematic review and meta-analysis of screening tests and interventions. *BMJ* 2017;356:i6538. doi: 10.1136/bmj.i6538
9. England PHEDUN. NHS Diabetes Prevention Programme, NHSDPP overview and FAQ *NHS England Publications* 2016(Gateway Reference 05728)
10. England PH. NHS Diabetes Prevention Programm (NHS DPP) Non-diabetic hyperglycaemia. In: (NCVIN) NCIN, ed. London: PHE publications, 2015.
11. Herrett E, Gallagher AM, Bhaskaran K, et al. Data Resource Profile: Clinical Practice Research Datalink (CPRD). *International journal of epidemiology* 2015;44(3):827-36. doi: 10.1093/ije/dyv098 [published Online First: 2015/06/08]
12. McLennan; D, Barnes; H, Noble; M, et al. The English indices of deprivation 2010. Online First: Oxford Institute of Social Policy, University of Oxford, 2011.
13. Springate DA, Kontopantelis E, Ashcroft DM, et al. ClinicalCodes: An Online Clinical Codes Repository to Improve the Validity and Reproducibility of Research Using Electronic Medical Records. *PLoS ONE* 2014;9(6):e99825. doi: 10.1371/journal.pone.0099825
14. Olier I, Springate DA, Ashcroft DM, et al. Modelling Conditions and Health Care Processes in Electronic Health Records: An Application to Severe Mental Illness with the Clinical Practice Research Datalink. *PLoS One* 2016;11(2):e0146715. doi: 10.1371/journal.pone.0146715 [published Online First: 2016/02/27]
15. Khan NF, Perera R, Harper S, et al. Adaptation and validation of the Charlson Index for Read/OXMIS coded databases. *BMC family practice* 2010;11:1. doi: 10.1186/1471-2296-11-1 [published Online First: 2010/01/07]
16. Charlson ME, Pompei P, Ales KL, et al. A new method of classifying prognostic comorbidity in longitudinal studies: development and validation. *Journal of chronic diseases* 1987;40(5):373-83. [published Online First: 1987/01/01]
17. CPRD @ Cambridge – Codes Lists Version 1.1 October 2018 [Available from: https://www.phpc.cam.ac.uk/pcu/cprd_cam/codelists/v11/ accessed 26/11/2018 2018.
18. Tate AR, Dungey S, Glew S, et al. Quality of recording of diabetes in the UK: how does the GP's method of coding clinical data affect incidence estimates? Cross-sectional study using the CPRD database. *BMJ open* 2017;7(1) doi: 10.1136/bmjopen-2016-012905
19. de Groot M, Anderson R, Freedland KE, et al. Association of Depression and Diabetes Complications: A Meta-Analysis. *Psychosomatic Medicine* 2001;63(4):619-30.
20. Nowakowska M, Zghebi SS, Ashcroft DM, et al. The comorbidity burden of type 2 diabetes mellitus: patterns, clusters and predictions from a large English primary care cohort. *BMC*

For peer review only - http://bmjopen.bmj.com/site/about/guidelines.xhtml

medicine 2019;17(1):145. doi: 10.1186/s12916-019-1373-y [published Online First: 2019/07/28]
21. Morris DH, Khunti K, Achana F, et al. Progression rates from HbA1c 6.0-6.4% and other prediabetes definitions to type 2 diabetes: a meta-analysis. *Diabetologia* 2013;56(7):1489-93. doi: 10.1007/s00125-013-2902-4 [published Online First: 2013/04/16]
22. Magliano DJ, Barr EL, Zimmet PZ, et al. Glucose indices, health behaviors, and incidence of diabetes in Australia: the Australian Diabetes, Obesity and Lifestyle Study. *Diabetes care* 2008;31(2):267-72. doi: 10.2337/dc07-0912 [published Online First: 2007/11/09]
23. Harris MI. Impaired glucose tolerance--prevalence and conversion to NIDDM. *Diabetic medicine : a journal of the British Diabetic Association* 1996;13(3 Suppl 2):S9-11. [published Online First: 1996/01/01]
24. Sharma M, Nazareth I, Petersen I. Trends in incidence, prevalence and prescribing in type 2 diabetes mellitus between 2000 and 2013 in primary care: a retrospective cohort study. *BMJ open* 2016;6(1):e010210. doi: 10.1136/bmjopen-2015-010210 [published Online First: 2016/01/16]
25. (WHO) WHO. Use of glycated haemoglobin (HbA1c) in the diagnosis of diabetes mellitus, 2011:25.
26. Katikireddi SV, Morling JR, Bhopal R. Is there a divergence in time trends in the prevalence of impaired glucose tolerance and diabetes? A systematic review in South Asian populations. *International journal of epidemiology* 2011;40(6):1542-53. doi: 10.1093/ije/dyr159
27. Mohan V, Deepa M, Deepa R, et al. Secular trends in the prevalence of diabetes and impaired glucose tolerance in urban South India—the Chennai Urban Rural Epidemiology Study (CURES-17). *Diabetologia* 2006;49(6):1175-78. doi: 10.1007/s00125-006-0219-2
28. Tabák AG, Herder C, Rathmann W, et al. Prediabetes: A high-risk state for developing diabetes. *Lancet* 2012;379(9833):2279-90. doi: 10.1016/S0140-6736(12)60283-9
29. DeFronzo RA, Tripathy D, Schwenke DC, et al. Pioglitazone for diabetes prevention in impaired glucose tolerance. *The New England journal of medicine* 2011;364(12):1104-15. doi: 10.1056/NEJMoa1010949 [published Online First: 2011/03/25]
30. (NICE) NIfHaCE. Type 2 diabetes: prevention in people at high risk, 2017:1-42.
31. Hong J-L, McNeill AM, He J, et al. Identification of impaired fasting glucose, healthcare utilization and progression to diabetes in the UK using the Clinical Practice Research Datalink (CPRD). *Pharmacoepidemiology and Drug Safety* 2016;25(12):1375-86. doi: doi:10.1002/pds.4007
32. Wang H, Shara NM, Calhoun D, et al. Incidence rates and predictors of diabetes in those with prediabetes: the Strong Heart Study. *Diabetes/metabolism research and reviews* 2010;26(5):378-85. doi: 10.1002/dmrr.1089
33. Edelstein SL, Knowler WC, Bain RP, et al. Predictors of progression from impaired glucose tolerance to NIDDM: an analysis of six prospective studies. *Diabetes* 1997;46(4):701-10.
34. Ben Haider NY, Ziyab AH. Prevalence of prediabetes and its association with obesity among college students in Kuwait: A cross-sectional study. *Diabetes Research and Clinical Practice* 2016;119:71-74. doi: https://doi.org/10.1016/j.diabres.2016.07.001
35. Tudor Hart J. THE INVERSE CARE LAW. *The Lancet* 1971;297(7696):405-12. doi: 10.1016/S0140-6736(71)92410-X

For peer review only - http://bmjopen.bmj.com/site/about/guidelines.xhtml

Acknowledgments

This manuscript is independent research funded by the National Institute for Health Research (Health Services and Delivery Research, 16/48/07 – Evaluating the NHS Diabetes Prevention Programme (NHS DPP): the DIPLOMA research programme (Diabetes Prevention – Long Term Multimethod Assessment)).

The views and opinions expressed in this manuscript are those of the authors and do not necessarily reflect those of the NHS, the National Institute for Health Research or the Department of Health and Social Care.

Copyright

The Corresponding Author has the right to grant on behalf of all authors and does grant on behalf of all authors, a worldwide licence to the Publishers and its licensees in perpetuity, in all forms, formats and media (whether known now or created in the future), to i) publish, reproduce, distribute, display and store the Contribution, ii) translate the Contribution into other languages, create adaptations, reprints, include within collections and create summaries, extracts and/or, abstracts of the Contribution, iii) create any other derivative work(s) based on the Contribution, iv) to exploit all subsidiary rights in the Contribution, v) the inclusion of electronic links from the Contribution to third party material where-ever it may be located; and, vi) licence any third party to do any or all of the above.

Declaration of competing interests

National Institute for Health Research (Health Services and Delivery Research, 16/48/07 – Evaluating the NHS Diabetes Prevention Programme (NHS DPP): the DIPLOMA research programme (Diabetes Prevention – Long Term Multimethod Assessment)). Funded the time and facilities of RR. SH contributes for consultancy for Eli Lilly, NovoNordisk, Takeda, Sanofi Aventis, Zealand Pharma, UN-EEG and is also part of the speakers panel for NovoNordisk. No other relationships or activities that could appear to have influenced the submitted work.

Authorship & contributorship

EK & RR designed the study, RR extracted the data from all sources and performed the analyses. RR wrote the manuscript. DR, EH, RM, SRC, SC, WW, SH, MS, PB and EK critically revised the manuscript. RR is the guarantor of this work and, as such, had full access to all the data in the study and takes responsibility for the integrity of the data and the accuracy of the data analysis.

Transparency declaration

RR affirms that this manuscript is an honest, accurate, and transparent account of the study being reported; that no important aspects of the study have been omitted; and that any discrepancies from the study as planned have been explained.

Data sharing

The data used in this study cannot be shared due to licencing restrictions by CPRD.

For peer review only - http://bmjopen.bmj.com/site/about/guidelines.xhtml

Dissemination Declaration

Not applicable

Ethical approval

The protocol for this study received scientific and ethical approval from the Independent Scientific Advisory Committee for CPRD studies (ISAC Protocol 18_101).

Patient involvement

Not applicable

For peer review only

For peer review only - http://bmjopen.bmj.com/site/about/guidelines.xhtml

Figure 1: Flow diagram of patient inclusions

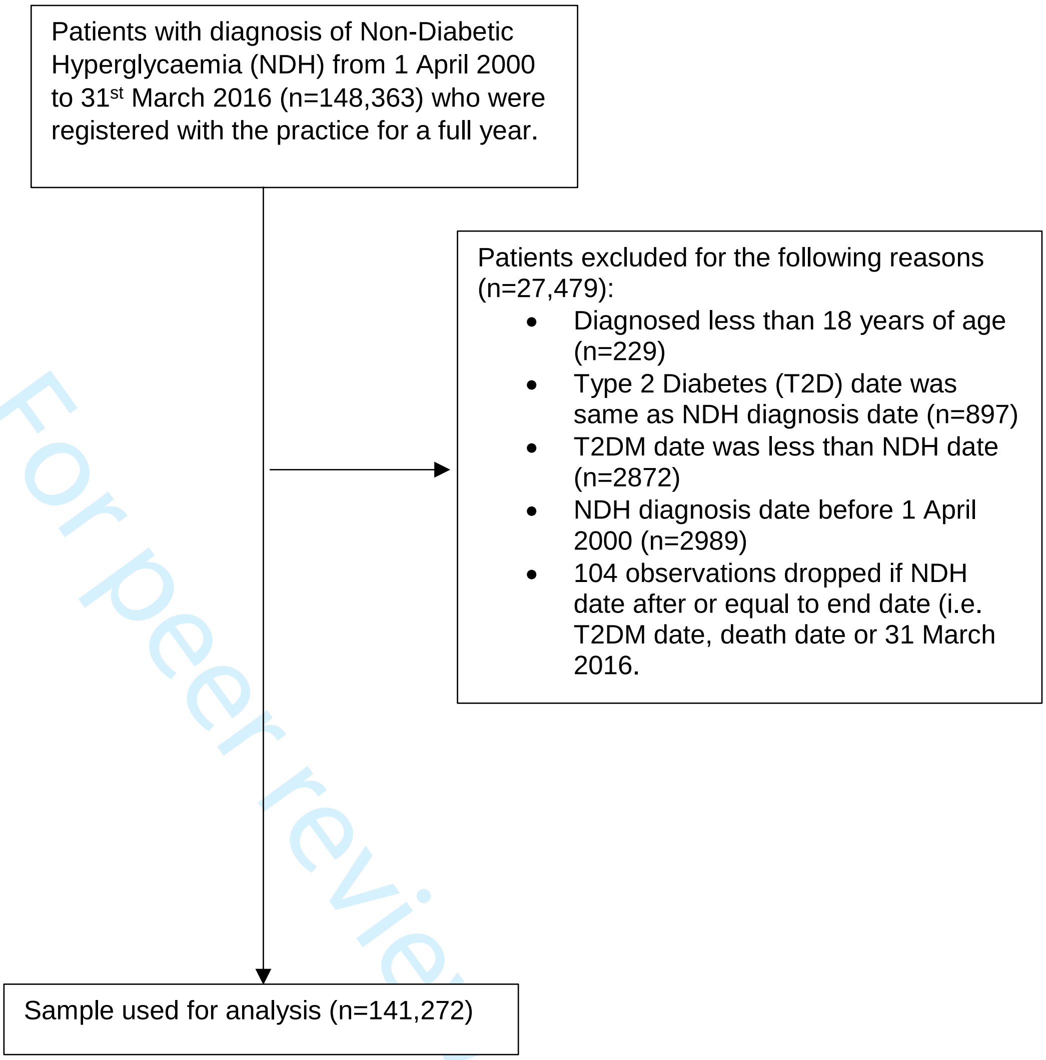

For peer review only

Figure 2: Cumulative conversion of NDH to type-2 diabetes from 2000 till 2015

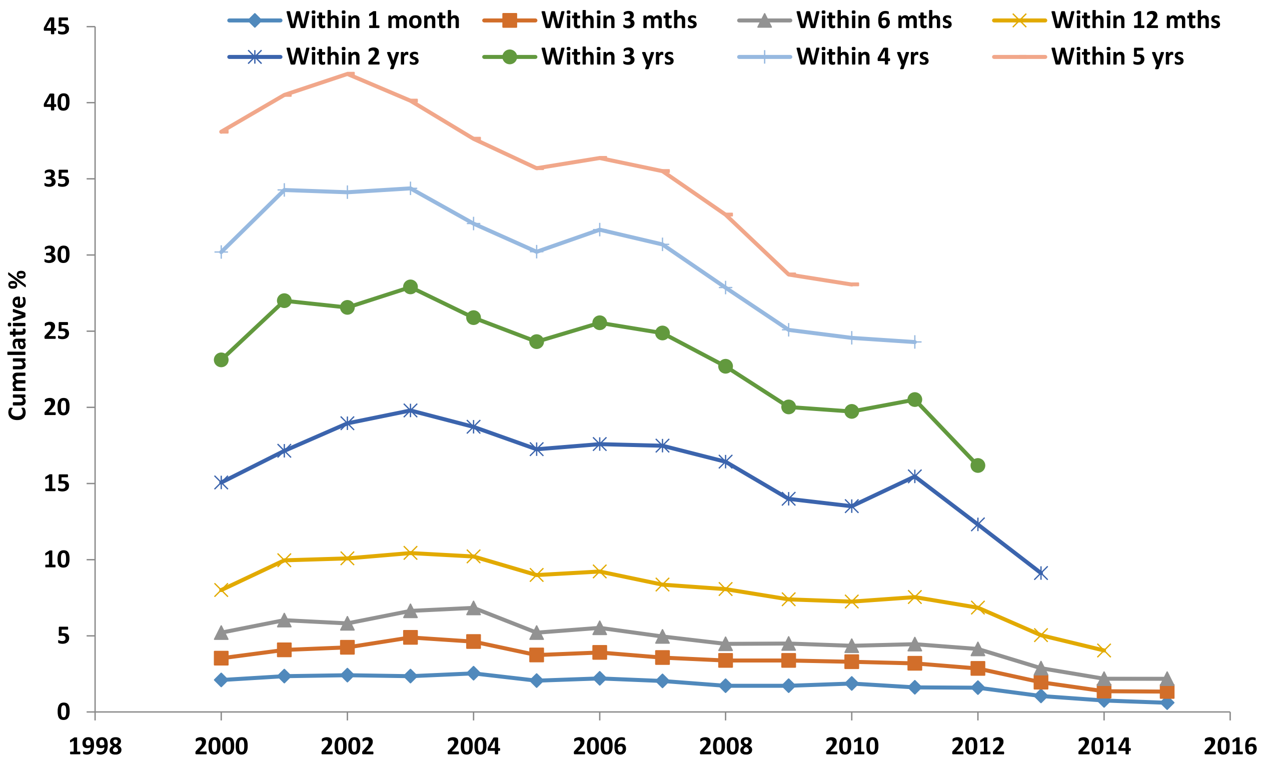

Note: Year 2000 defined as 01st April 2000 till 31st March 2001 and other years defined similarly

review only

Supplementary

Table 1: Read codes used to diagnose Type 2 Diabetes Mellitus

Medcode	Readcode	Description
506	C100112	Non-insulin dependent diabetes mellitus
758	C10F.00	Type 2 diabetes mellitus
1407	C10FJ00	Insulin treated Type 2 diabetes mellitus
4513	C109.00	Non-insulin dependent diabetes mellitus
5884	C109.11	NIDDM - Non-insulin dependent diabetes mellitus
8403	C109700	Non-insulin dependent diabetes mellitus - poor control
12640	C10FC00	Type 2 diabetes mellitus with nephropathy
12736	C10F500	Type 2 diabetes mellitus with gangrene
17262	C109600	Non-insulin-dependent diabetes mellitus with retinopathy
17859	C109.12	Type 2 diabetes mellitus
18143	C109G11	Type II diabetes mellitus with arthropathy
18209	C109012	Type 2 diabetes mellitus with renal complications
18219	C109.13	Type II diabetes mellitus
18264	C109J12	Insulin treated Type II diabetes mellitus
18278	C109J00	Insulin treated Type 2 diabetes mellitus
18390	C10FM00	Type 2 diabetes mellitus with persistent microalbuminuria
18425	C10FB00	Type 2 diabetes mellitus with polyneuropathy
18496	C10F600	Type 2 diabetes mellitus with retinopathy
18777	C10F000	Type 2 diabetes mellitus with renal complications
22884	C10F.11	Type II diabetes mellitus
24458	C109711	Type II diabetes mellitus - poor control
24693	C109G00	Non-insulin dependent diabetes mellitus with arthropathy
24836	C109C12	Type 2 diabetes mellitus with nephropathy
25041	ZC2CA00	Dietary advice for type II diabetes
25591	C10FQ00	Type 2 diabetes mellitus with exudative maculopathy
25627	C10F700	Type 2 diabetes mellitus - poor control
26054	C10FL00	Type 2 diabetes mellitus with persistent proteinuria
29979	C109900	Non-insulin-dependent diabetes mellitus without complication
32627	C10FN00	Type 2 diabetes mellitus with ketoacidosis
34268	C10F200	Type 2 diabetes mellitus with neurological complications
34450	C10FK00	Hyperosmolar non-ketotic state in type 2 diabetes mellitus
34912	C109400	Non-insulin dependent diabetes mellitus with ulcer
35385	C10FH00	Type 2 diabetes mellitus with neuropathic arthropathy
36633	C109K00	Hyperosmolar non-ketotic state in type 2 diabetes mellitus
36695	C10D.00	Diabetes mellitus autosomal dominant type 2
37648	C109J11	Insulin treated non-insulin dependent diabetes mellitus
37806	C10FF00	Type 2 diabetes mellitus with peripheral angiopathy
40401	C109500	Non-insulin dependent diabetes mellitus with gangrene
42762	C109612	Type 2 diabetes mellitus with retinopathy
43227	C10F311	Type II diabetes mellitus with multiple complications
43785	C109D00	Non-insulin dependent diabetes mellitus with hypoglyca coma
44779	C109E12	Type 2 diabetes mellitus with diabetic cataract
44982	C10FE00	Type 2 diabetes mellitus with diabetic cataract
45467	C109B00	Non-insulin dependent diabetes mellitus with polyneuropathy
45913	C109712	Type 2 diabetes mellitus - poor control
45919	C109212	Type 2 diabetes mellitus with neurological complications
46150	C109512	Type 2 diabetes mellitus with gangrene

For peer review only - http://bmjopen.bmj.com/site/about/guidelines.xhtml

Medcode	Readcode	Description
46917	C10FD00	Type 2 diabetes mellitus with hypoglycaemic coma
47315	C10F711	Type II diabetes mellitus - poor control
47321	C10F100	Type 2 diabetes mellitus with ophthalmic complications
47409	C109B11	Type II diabetes mellitus with polyneuropathy
47816	C109H11	Type II diabetes mellitus with neuropathic arthropathy
47954	C10F900	Type 2 diabetes mellitus without complication
48192	C109E11	Type II diabetes mellitus with diabetic cataract
49074	C10F400	Type 2 diabetes mellitus with ulcer
49655	C10F611	Type II diabetes mellitus with retinopathy
49869	C109G12	Type 2 diabetes mellitus with arthropathy
50225	C109011	Type II diabetes mellitus with renal complications
50429	C109100	Non-insulin-dependent diabetes mellitus with ophthalm comps
50527	C10FB11	Type II diabetes mellitus with polyneuropathy
50609	L180600	Pre-existing diabetes mellitus, non-insulin-dependent
50813	C109A11	Type II diabetes mellitus with mononeuropathy
51756	C10FP00	Type 2 diabetes mellitus with ketoacidotic coma
52303	C109000	Non-insulin-dependent diabetes mellitus with renal comps
53392	C10F911	Type II diabetes mellitus without complication
54899	C109F11	Type II diabetes mellitus with peripheral angiopathy
55075	C109411	Type II diabetes mellitus with ulcer
55842	C109200	Non-insulin-dependent diabetes mellitus with neuro comps
56268	C109D11	Type II diabetes mellitus with hypoglycaemic coma
56803	C107400	NIDDM with peripheral circulatory disorder
57278	C10F011	Type II diabetes mellitus with renal complications
58604	C109611	Type II diabetes mellitus with retinopathy
59253	C10FG00	Type 2 diabetes mellitus with arthropathy
59365	C109C00	Non-insulin dependent diabetes mellitus with nephropathy
59725	C109111	Type II diabetes mellitus with ophthalmic complications
60699	C109F12	Type 2 diabetes mellitus with peripheral angiopathy
60796	C10FL11	Type II diabetes mellitus with persistent proteinuria
61071	C109D12	Type 2 diabetes mellitus with hypoglycaemic coma
62107	C109511	Type II diabetes mellitus with gangrene
62146	C109300	Non-insulin-dependent diabetes mellitus with multiple comps
62674	C10FA00	Type 2 diabetes mellitus with mononeuropathy
63690	C10FR00	Type 2 diabetes mellitus with gastroparesis
64571	C109C11	Type II diabetes mellitus with nephropathy
64668	C10FJ11	Insulin treated Type II diabetes mellitus
65267	C10F300	Type 2 diabetes mellitus with multiple complications
65704	C109412	Type 2 diabetes mellitus with ulcer
66965	C109H12	Type 2 diabetes mellitus with neuropathic arthropathy
67905	C109211	Type II diabetes mellitus with neurological complications
69278	C109E00	Non-insulin depend diabetes mellitus with diabetic cataract
70316	C109112	Type 2 diabetes mellitus with ophthalmic complications
72320	C109A00	Non-insulin dependent diabetes mellitus with mononeuropathy
83532	66Ao.00	Diabetes type 2 review
85991	C10FM11	Type II diabetes mellitus with persistent microalbuminuria
91646	C10F411	Type II diabetes mellitus with ulcer
93727	C10FE11	Type II diabetes mellitus with diabetic cataract
95351	C10FA11	Type II diabetes mellitus with mononeuropathy

For peer review only - http://bmjopen.bmj.com/site/about/guidelines.xhtml

Medcode	Readcode	Description
98616	C10F211	Type II diabetes mellitus with neurological complications
98723	C10FD11	Type II diabetes mellitus with hypoglycaemic coma
100964	C10F111	Type II diabetes mellitus with ophthalmic complications
101801	66At100	Type II diabetic dietary review
102201	C10FC11	Type II diabetes mellitus with nephropathy
102611	66At111	Type 2 diabetic dietary review
103902	C10FG11	Type II diabetes mellitus with arthropathy
104323	C10F511	Type II diabetes mellitus with gangrene
104639	C10FF11	Type II diabetes mellitus with peripheral angiopathy
105784	C109912	Type 2 diabetes mellitus without complication
106061	C10FP11	Type II diabetes mellitus with ketoacidotic coma
106528	C10FN11	Type II diabetes mellitus with ketoacidosis
107701	C10FK11	Hyperosmolar non-ketotic state in type II diabetes mellitus
108005	C109312	Type 2 diabetes mellitus with multiple complications
109103	C109911	Type II diabetes mellitus without complication
109197	C10FH11	Type II diabetes mellitus with neuropathic arthropathy
109865	C109B12	Type 2 diabetes mellitus with polyneuropathy
111798	C10FQ11	Type II diabetes mellitus with exudative maculopathy

For peer review only

STROBE Statement—Checklist of items that should be included in reports of ***cohort studies***

	Item No	Recommendation	Page No
**Title and abstract**	1	(*a*) Indicate the study's design with a commonly used term in the title or the abstract	1-2
		(*b*) Provide in the abstract an informative and balanced summary of what was done and what was found	2
**Introduction**			
Background/rationale	2	Explain the scientific background and rationale for the investigation being reported	3
Objectives	3	State specific objectives, including any prespecified hypotheses	3-4
**Methods**			
Study design	4	Present key elements of study design early in the paper	4
Setting	5	Describe the setting, locations, and relevant dates, including periods of recruitment, exposure, follow-up, and data collection	4
Participants	6	(*a*) Give the eligibility criteria, and the sources and methods of selection of participants. Describe methods of follow-up	4
		(*b*) For matched studies, give matching criteria and number of exposed and unexposed	
Variables	7	Clearly define all outcomes, exposures, predictors, potential confounders, and effect modifiers. Give diagnostic criteria, if applicable	5
Data sources/ measurement	8*	For each variable of interest, give sources of data and details of methods of assessment (measurement). Describe comparability of assessment methods if there is more than one group	5
Bias	9	Describe any efforts to address potential sources of bias	5
Study size	10	Explain how the study size was arrived at	4
Quantitative variables	11	Explain how quantitative variables were handled in the analyses. If applicable, describe which groupings were chosen and why	5-6
Statistical methods	12	(*a*) Describe all statistical methods, including those used to control for confounding	7
		(*b*) Describe any methods used to examine subgroups and interactions	
		(*c*) Explain how missing data were addressed	
		(*d*) If applicable, explain how loss to follow-up was addressed	
		(*e*) Describe any sensitivity analyses	
**Results**			
Participants	13*	(a) Report numbers of individuals at each stage of study—eg numbers potentially eligible, examined for eligibility, confirmed eligible, included in the study, completing follow-up, and analysed	5-6 Figure 1 (PDF)
		(b) Give reasons for non-participation at each stage	
		(c) Consider use of a flow diagram	
Descriptive data	14*	(a) Give characteristics of study participants (eg demographic, clinical, social) and information on exposures and potential confounders	12 Table 3 (Page 15)
		(b) Indicate number of participants with missing data for each variable of interest	
		(c) Summarise follow-up time (eg, average and total amount)	

For peer review only

Outcome data	15*	Report numbers of outcome events or summary measures over time	5-6

For peer review only

For peer review only - http://bmjopen.bmj.com/site/about/guidelines.xhtml

Main results	16	(*a*) Give unadjusted estimates and, if applicable, confounder-adjusted estimates and their precision (eg, 95% confidence interval). Make clear which confounders were adjusted for and why they were included<br>(*b*) Report category boundaries when continuous variables were categorized<br>(*c*) If relevant, consider translating estimates of relative risk into absolute risk for a meaningful time period	7-16
Other analyses	17	Report other analyses done—eg analyses of subgroups and interactions, and sensitivity analyses	
**Discussion**			
Key results	18	Summarise key results with reference to study objectives	17
Limitations	19	Discuss limitations of the study, taking into account sources of potential bias or imprecision. Discuss both direction and magnitude of any potential bias	17
Interpretation	20	Give a cautious overall interpretation of results considering objectives, limitations, multiplicity of analyses, results from similar studies, and other relevant evidence	20
Generalisability	21	Discuss the generalisability (external validity) of the study results	17
**Other information**			
Funding	22	Give the source of funding and the role of the funders for the present study and, if applicable, for the original study on which the present article is based	24

*Give information separately for exposed and unexposed groups.

Note: An Explanation and Elaboration article discusses each checklist item and gives methodological background and published examples of transparent reporting. The STROBE checklist is best used in conjunction with this article (freely available on the Web sites of PLoS Medicine at http://www.plosmedicine.org/, Annals of Internal Medicine at http://www.annals.org/, and Epidemiology at http://www.epidem.com/). Information on the STROBE Initiative is available at http://www.strobe-statement.org.

For peer review only

For peer review only - http://bmjopen.bmj.com/site/about/guidelines.xhtml

BMJ Open

The epidemiology and determinants of non-diabetic hyperglycaemia and its conversion to type 2 diabetes mellitus, 2000-2015: cohort population study using UK electronic health records.

Journal:	*BMJ Open*
Manuscript ID	bmjopen-2020-040201.R1
Article Type:	Original research
Date Submitted by the Author:	14-Jul-2020
Complete List of Authors:	Ravindrarajah, Rathi; The University of Manchester, Faculty of Biology, Medicine and Health;<br>Reeves, David; University of Manchester, Institute of Population Health; Centre for Biostatistics<br>Howarth, Elizabeth; The University of Manchester, Faculty of Biology, Medicine and Health<br>Meacock, Rachel; Univ Manchester,<br>Soiland-Reyes, Claudia; Salford Royal NHS Foundation Trust; Salford Royal NHS Foundation Trust<br>Cotterill, Sarah; University of Manchester, Centre for Biostatistics<br>Whittaker, William; University of Manchester, Manchester Centre for Health Economics<br>Heller, Simon; Unversity of Sheffield, Academic Unit of Diabetes, Endo and Metab<br>Sutton, Matt; University of Manchester<br>Bower, Peter; University of Manchester, NPCRDC<br>Kontopantelis, Evangelos; The University of Manchester, Faculty of Biology, Medicine and Health
**Primary Subject Heading**:	Epidemiology
Secondary Subject Heading:	Public health
Keywords:	DIABETES & ENDOCRINOLOGY, EPIDEMIOLOGY, PRIMARY CARE

SCHOLARONE™
Manuscripts

I, the Submitting Author has the right to grant and does grant on behalf of all authors of the Work (as defined in the below author licence), an exclusive licence and/or a non-exclusive licence for contributions from authors who are: i) UK Crown employees; ii) where BMJ has agreed a CC-BY licence shall apply, and/or iii) in accordance with the terms applicable for US Federal Government officers or employees acting as part of their official duties; on a worldwide, perpetual, irrevocable, royalty-free basis to BMJ Publishing Group Ltd ("BMJ") its licensees and where the relevant Journal is co-owned by BMJ to the co-owners of the Journal, to publish the Work in this journal and any other BMJ products and to exploit all rights, as set out in our licence.

The Submitting Author accepts and understands that any supply made under these terms is made by BMJ to the Submitting Author unless you are acting as an employee on behalf of your employer or a postgraduate student of an affiliated institution which is paying any applicable article publishing charge ("APC") for Open Access articles. Where the Submitting Author wishes to make the Work available on an Open Access basis (and intends to pay the relevant APC), the terms of reuse of such Open Access shall be governed by a Creative Commons licence – details of these licences and which Creative Commons licence will apply to this Work are set out in our licence referred to above.

Other than as permitted in any relevant BMJ Author's Self Archiving Policies, I confirm this Work has not been accepted for publication elsewhere, is not being considered for publication elsewhere and does not duplicate material already published. I confirm all authors consent to publication of this Work and authorise the granting of this licence.

For peer review only

The epidemiology and determinants of non-diabetic hyperglycaemia and its conversion to type 2 diabetes mellitus, 2000-2015: cohort population study using UK electronic health records.

Rathi Ravindrarajah[a,], David Reeves[a] ,Elizabeth Howarth [a,], Rachel Meacock[a], Soiland-Reyes Claudia[c], Sarah Cotterill[b], William Whittaker[a], Simon Heller[d], Matt Sutton[a], Peter Bower[a], Evangelos Kontopantelis[a]

[a] Division of Population Health, Faculty of Biology, Medicine and Health, University of Manchester
[b] Centre for Biostatistics, School of Health Sciences, University of Manchester
[c] NIHR CLAHRC Greater Manchester, Salford Royal NHS Foundation Trust, Salford, UK
[d] Dept. of Oncology and Metabolism, University of Sheffield

Dr. David Reeves (PhD),Dr. Elizabeth Howarth (PhD), Dr.Rachel Meacock (PhD), Ms. Soiland-Reyes Claudia (MPhil), Dr. Sarah Cotterill (PhD), Dr. William Whittaker (PhD), Pof. Simon Heller (FRCP), Prof. Matt Sutton (PhD), Prof. Peter Bower (PhD), Prof. Evan Kontopantelis (PhD)

Corresponding Author: Rathi Ravindrarajah (PhD),
Research Associate
Division of Population Health
Faculty of Biology, Medicine and Health
University of Manchester
Email id: rathi.ravindrarajah@manchester.ac.uk

For peer review only

Word Count: 4038

Key Words: Non-Diabetic Hyperglycaemia, Prediabetes, Electronic Health Records, Type 2 Diabetes

For peer review only - http://bmjopen.bmj.com/site/about/guidelines.xhtml

Abstract

Objectives

To study the characteristics of UK individuals identified with non-diabetic hyperglycaemia (NDH) and their conversion rates to Type 2 Diabetes Mellitus (T2DM) from 2000 to 2015, using the Clinical Practice Research Datalink (CPRD).

Design

Cohort study

Settings

UK primary Care Practices

Participants

Electronic health records identified 14,272 participants with NDH, from 2000 to 2015

Primary and Secondary Outcome Measures

Baseline characteristics and conversion trends from NDH to T2DM were explored. Cox proportional-hazards models evaluated predictors of conversion.

Results

Crude conversion was 4% within 6 months of NDH diagnosis, 7% annually, 13% within 2 years, 17% within 3 years and 23% within 5 years. However, 1-year conversion fell from 8% in 2000 to 4% in 2014. Individuals aged 45-54 were at the highest risk of developing T2DM (HR= 1.20; 95% CI: 1.15, 1.25 – compared to those aged 18-44), and the risk reduced with older age. A BMI above 30 kg/m^2 was strongly associated with conversion (HR=2.02; 95% CI: 1.92, 2.13 – compared to those with a normal BMI). Depression (HR=1.10; 95% CI: 1.07, 1.13), smoking (HR=1.07; 95% CI: 1.03, 1.11 – compared to non-smokers) or residing in the most deprived areas (HR=1.17; 95% CI: 1.11, 1.24 – compared to residents of the most affluent areas) was modestly associated with conversion.

Conclusion

Although the rate of conversion from NDH to T2DM fell between 2010 and 2015, this is likely due to changes over time in the cut-off points for defining NDH, and more people of lower diabetes risk being diagnosed with NDH over time. People aged 45-54, smokers, depressed, with high BMI, and more deprived are at increased risk of conversion to T2DM.

Funding

This manuscript is independent research funded by the National Institute for Health Research (Health Services and Delivery Research, 16/48/07 – Evaluating the NHS Diabetes Prevention Programme (NHS DPP): the DIPLOMA research programme (Diabetes Prevention – Long Term Multimethod Assessment)).

For peer review only - http://bmjopen.bmj.com/site/about/guidelines.xhtml

Strengths and limitations of the Study

- Data was based on a large, anonymised, longitudinal and nationally representative sample of general practices
- The length of the study period (2000 to 2015) was useful in capturing changes over time
- Cases of NDH and T2DM were identified using Read codes, and the quality of recording may have been problematic for the former in earlier years
- Our NDH code list included a few relevant items and is not sensitive to misclassification

For peer review only

For peer review only - http://bmjopen.bmj.com/site/about/guidelines.xhtml

Introduction

The proportion of the population with type-2 diabetes mellitus (T2DM) has been rising globally and is an important contributor to mortality, morbidity and health care costs. It has been estimated that 415m people live with diabetes across the globe and 193m people have undiagnosed diabetes [1]. It has been suggested that currently there are 5 million people in England who are at risk of developing T2DM [2]. T2DM is characterized by pancreatic dysfunction causing insulin resistance. There are other key pathophysiological processes which increase the risk of T2DM, which involves organs including pancreas, liver, skeletal muscle, kidneys, brain, small intestine and adipose tissue[3]. Lifestyle factors such as excess weight and physical inactivity are known to increase the risk of developing T2DM.

Non-diabetic hyperglycaemia (NDH also known as pre-diabetes or impaired glucose regulation), refers to levels of blood glucose that are increased from the normal range but not yet high enough to be in the diabetic range. Previous research has shown that individuals diagnosed with NDH are at a higher risk of developing T2DM [4]. The NHS RightCare diabetes pathway defines NDH as having an HbA1c measurement in the 42-47 mmol/mol range (6.0-6.4%), or fasting plasma glucose in the 5.5-6.9 mmol/mol range [5]. Previous analyses using Health Survey England data have shown discrepancies in the prevalence of NDH in the UK. While one study suggested that the average NDH prevalence was 11% in adults aged 16+ in England, in the period between 2009 and 2013 [6], the other suggested a sharp rise in the prevalence of NDH from 11.6% in 2003 to 35.3% in 2011 in all adults [7]. The use of different cut-points for HbA1C used to define NDH has been suggested as the cause of this discrepancy; one study used the NICE and Diabetes UK cut-points (HbA1C: 42-47 mmol/mol) whereas the other used the American Diabetes Association cut-points (HbA1C: 39-47 mmol/mol). Delaying or preventing T2DM has become an international priority due to the burden that the condition places on both patients and health services [8]. NHS England, Public Health England and Diabetes UK have implemented a programme to identify those at high risk of developing T2DM and offer them an evidence-based behavioural intervention (NHS Diabetes Prevention Programme: NHS DPP) to people identified as having NDH in an attempt to reduce the incidence of T2DM and the complications related to it [9].

This paper explores two aspects of the epidemiology of people diagnosed with NDH in UK primary care. First, we aimed to estimate the prevalence of NDH and to explore the characteristics of patients with NDH in a population cohort of adults from 2000 until 2015. We chose this study period both to ensure high data quality and to avoid introducing bias into our analysis from any potential effects from the National Diabetes Prevention Programme [10]. Second, we evaluated the conversion rates of NDH

For peer review only - http://bmjopen.bmj.com/site/about/guidelines.xhtml

to T2DM over time, and whether conversion rates differ by age, sex, BMI levels, depression, multi-morbidity and area level deprivation.

Methods

Data Source

Patient level data was obtained from the Clinical Practice Research Datalink (CPRD), one of the largest active primary care databases of electronic health records (EHR) in the UK [11]. This dataset captures approximately 7% of the total UK population. The database holds anonymised data which contains information on clinical signs, diagnoses, tests and procedures [11]. Approximately 60% of all UK CPRD practices participate in the CPRD linkage scheme, which provides additional patient-level information. For this work, we obtained patient-level deprivation through the Office of National Statistics (ONS) linkage, in the form of the 2010 Index of Multiple Deprivation (IMD) [12].

Study Participants

Practices taking part in the CPRD are checked for eligibility in each year using a CPRD assessment algorithm, and evaluated to be of research standard or not. Patients were regarded as eligible if they had been registered with a practice for a full year, were aged 18 years and over and had a code for NDH between 1st April 2000 and 31st March 2016. At least one relevant Read code was considered adequate to flag a patient. Codes were identified using a strategy that involved searching for relevant terms through an algorithm, with the returned list being reviewed and finalised by members of the research team, as described elsewhere [13 14]. Read codes which were actively used by GPs to identify NDH were included in the study: 44v2.00 (Glucose Tolerance Test impaired), C11y200 (Impaired glucose tolerance), C11y300 (Impaired fasting glycaemia), C11y500 (Pre-diabetes), C317.00 (Non-diabetic Hyperglycaemia), R102.00 ([D] Glucose Tolerance Test abnormal), R102.11 ([D] Prediabetes), R102.12 ([D] Impaired glucose tolerance test), R10D000 ([D] Impaired fasting glycaemia), R10D011 ([D] Impaired fasting glucose), R10E.00 ([D] Impaired glucose tolerance. Eligible patients were followed up until censored at the earliest of any of the following events: diagnosed with T2DM (the outcome event), transferred out of practice (any cause), last collection date for the practice, end date of the study (31st March 2016) or death. To report prevalence, we also included cases that were diagnosed with NDH at any point prior to 1st April 2000, who met all other inclusion criteria.

Study measures

We calculated the prevalence of NDH in each year between 2000 and 2015, and conversion to T2DM was also determined. People with at least one relevant Read code of T2DM following the NDH diagnosis (the index date), were considered to have progressed to T2DM during the study period

For peer review only - http://bmjopen.bmj.com/site/about/guidelines.xhtml

(Supplement Table 1 provides a list of read codes used to diagnose T2DM). Patients with a previous record of Type-1 Diabetes were excluded.

We extracted information on the following covariates which have previously been reported [10] to be relevant to NDH and T2DM; age, gender, BMI, total serum cholesterol, smoking status, socio economic status and depression. Age was grouped into the following bands: 18-34, 35-44, 45-54, 55-64, 65-74, 75-84, and 85 years or over. The latest available measurement before the NDH diagnosis date, up until the previous 12 months, was used to define baseline total cholesterol and BMI. If such a value was not available, the measurement was set to missing. BMI values were classified into the following categories: underweight (<18.5 kg/m^2), normal weight (18.5-24.9 kg/m^2), overweight (25.0-29.9 kg/m^2) and obese (>=30 kg/m^2). Total serum cholesterol in mmol/l was categorised into: under 3.0, [3.0, 4.0), [4.0, 5.0), [5.0, 6.0) and 6.0 or over. We also quantified the multi-morbidity burden, using the Charlson Comorbidity Index (CCI), which is a widely used measure which assigns different weights to different conditions and includes: any malignancy, cerebrovascular disease, chronic pulmonary disease, congestive cardiac disease, dementia, HIV/AIDS, hemiplegia, lymphoproliferative disorders, metastatic solid tumour, mild liver disease, moderate and severe liver disease (CCI also includes diabetes with complications, which we necessarily excluded)[15 16]. This modified CCI was calculated using the list of validated diagnostic primary care Read codes used by Khan et al [15]. Participants were classified as having a condition if the condition was present at diagnosis of NDH or 12 months prior to diagnosis of NDH. CCI takes integer values and was categorised as: 0, 1 to 2, 3 to 4 and greater than 4. Depression was evaluated using medical codes and therapy codes which were obtained from the code lists derived from the CPRD provided on a Cambridge University repository [17]. Participants were considered to have depression at the index date (the date of NDH diagnosis) if they were recorded as depressed either by a code or if they were on relevant medication in the last 12 months. Smoking status was determined from information in the patients' record and categorised as "smoker", "ex-smoker" or "never smoked". The Index of Multiple Deprivation (IMD) was used to classify deprivation and the IMD scores were divided into quintiles.

For peer review only

Conversion of NDH to Type 2 Diabetes Mellitus

The time of conversion of NDH to T2DM was defined as the time from the index date (diagnosis of NDH) to the date they were diagnosed as having T2DM. This time was then categorised into progression time of: 1 month; 3 months, 6 months, 12 months, 2 years, 3 years, 4 years, and 5 years. Those who had a conversion time of over 5 years were excluded from analysis. In addition, patients who did not convert to T2DM, left the study or died within this study period were categorised into a single category as "Not converted/left/died". A small number of participants were diagnosed as having T2DM on, or ever before, the index date, and were excluded from further analyses (See Figure 1).

For peer review only - http://bmjopen.bmj.com/site/about/guidelines.xhtml

Statistical Analysis

The characteristics of people identified with NDH are presented descriptively. Conversion rates of NDH to T2DM, in the progression time categories were plotted over time. Annual bins were defined as financial years, for example 1st April 2000 to 31st March 2001 was labelled as 2000. The associations between covariates and conversion from NDH to T2DM were estimated in a time to event analysis. A Cox proportional hazards model was employed to estimate adjusted hazard ratios (HRs) of the associations between conversion and the following covariates: gender, age groups, BMI categories, total cholesterol levels, depression, year, patient-level deprivation scores and CCI categories. Proportionality of hazards was tested using Schoenfeld residuals.

Patient involvement

CPRD data provides anonymised patient data hence patients are not identified by the researchers.

Results

Over the study period, a total of 148,363 participants were identified with NDH. The prevalence and incidence of NDH for each financial year is shown in Table 1. Prevalence increased from 0.07% in 2000 to 1.85% in 2015. Incidence of NDH increased from 0.02% in 2000 to 0.21% in 2015. Table 2 and Figure 2 show the cumulative frequency of conversion from NDH to T2DM, by year, from 1 April 2000 to 31 March 2016. Frequency of conversion within one financial year peaks in 2003 and then follows a decreasing trend. Amongst this general trend of declining conversion, there was a peak in the year 2011, with a further exploration of the data (results not shown) suggesting that patients had somewhat higher BMIs in this year, although that does not fully explain the rise.

After all exclusion criteria were applied (see Figure 1), our final NDH population was 141,272 people, with a mean follow-up period of 5 years since the index date.

Table 3 displays the baseline characteristics of the cohort. Covariates are treated as categorical variables in our analysis, and so reported here as numbers and percentages. The mean age of the cohort was 63.2 (SD=13.4) years, and 53% were male. The prevalence of NDH was highest in those aged 65-74 years (39,178/141,272; 27.7%). The proportion of NDH was higher in older females (3728/67,369, 5.5%), compared to older males (2162/73,903; 2.9%) aged 85 years and more. The most common BMI category in our cohort was obese, with 32% of females with a measurement of BMI equal to or above 30 kg/m^2. Results showed that 19% of the NDH cohort had depression when they were diagnosed with NDH. The vast majority of the NDH population (85%) had a Charlson comorbidity score of zero at the index date, indicating absence of major comorbidities.

For peer review only - http://bmjopen.bmj.com/site/about/guidelines.xhtml

Table 4 shows the number of patients who converted from NDH to T2DM. Over the whole of the study period, the conversion rates were: 1.6% within 1 month, 3% within 3 months, 4.2% within 6 months, 7% within a year, 12.8% within 24 months, 17.2% within 3 years, 20.4% within 4 years and 22.8% over 5 years. The majority (77.2%, n=104,030) did not convert, but the length of time each was followed up varied depending on the time they were diagnosed with NDH.

Table 5 shows the results from the Cox proportional hazard models, which explored time to conversion from NDH to T2DM, with failure being the diagnosis of T2DM. Residuals were linear over time, indicating that proportionality generally stood. The rate of conversion was highest for the 45-54 age-group with HR=1.20 (95% CI 1.15 to 1.25), compared to those aged 18-44, and the risk steadily decreased with increasing age to a HR of 0.65 (95% CI 0.60 to 0.71) for people aged 85 or over. Cholesterol categories did not appear to be strongly associated with conversion to T2DM. People with high BMI had a much higher risk of conversion to T2DM, with those classed overweight (BMI 25-30) having a HR of 1.40 (95% CI: 1.33 to 1.48), and those classed obese (BMI>=30) having a HR of 2.0 (95% CI: 1.9, 2.1), compared to individuals with a normal BMI (18.5 to 25). Compared to non- smokers, current smokers had a slightly increased risk of converting to T2DM with a HR of 1.07 (95% CI of 1.03 to 1.11). Those who had a CCI score of 1 to 2 had a slightly higher risk of conversion to T2DM with a HR of 1.1 (95% CI: 1.08 to 1.15) but there was no increased risk among those with higher CCI scores. Having depression at baseline slightly increased the risk of conversion (HR=1.10, 95% CI 1.07, 1.13). The risk of conversion to T2DM increased with patient level deprivation as measured by the 2010 IMD, suggesting that those living in more deprived areas are at an increased risk of conversion from NDH to T2DM. Patients living in the least affluent quintile had an HR of 1.17 (95% CI 1.11 to 1.24), compared to patients living in the most affluent quintile.

Discussion

In our cohort, incidence of NDH increased from 0.02% in 2000 to 0.21% in 2015. NDH is more common in males and the proportion with NDH increased with age, up to 75 years. The proportion of individuals diagnosed with NDH increased with BMI. The time taken to convert from NDH to T2DM was further explored which showed that approximately 7% converted to T2DM within a year. The conversion rates were also explored by year from 2000 till 2015, which showed a general trend of a decline in the conversion rate from NDH to T2DM with a peak in the year 2004 and 2011. The risk of conversion from NDH to T2DM was higher in men and those aged 45 to 54 years, decreasing with age. People with NDH who are overweight, and even more so those who are obese, have a higher risk of developing diabetes. Depression, deprivation and smoking (perhaps as a deprivation proxy) were also modestly associated with T2DM conversion.

For peer review only - http://bmjopen.bmj.com/site/about/guidelines.xhtml

Our study has several strengths. It was based on a large, longitudinal and nationally representative data resource. The length of the study period is also useful in capturing changes over time. This study has some limitations. Our diagnosed cases of NDH and T2DM are based on Read codes being used. Although we could have considered other approaches to define NDH and T2DM to avoid false positives, in the context of the UK primary care, coding of T2DM is known to be of very high quality because of the Quality and Outcomes Framework (QOF), which incentive GPs for accurate recording[14]. Although this change occurred in 2004, quality was already high from 2000 onwards, in anticipation for the scheme and other smaller-scale frameworks. The only potential issue with the QOF was the non-distinction in coding between Type-1 and Type-2, until explicitly requested in 2006. This may have led to us missing a few cases that exited the database before 2006, if they had type-2 diabetes but were only given a generic diabetes code. In our experience this is very rare, however and it would not affect our finding that conversion rates for NDH have dropped over time. As previously mentioned, the quality of recording is very high and people associated with a Read code for T2DM, have the condition – there is no provisional coding and GPs are encouraged to add to records only if certain since they know retracting such a diagnosis is very complicated. If someone is suspected of having the condition they will be not be given a Read code, but information will be added in notes (or with a "suspected diabetes" code). Remission is possible of course, although rare, but it is not relevant for this study (where T2DM is the outcome of interest in a time to event analysis).

Regarding NDH coding, the situation is more complicated because of the absence of financial incentives through the QOF, hence practice variability is greater. In addition, the definition of NDH has changed over time, as we explain in the paper, making it difficult to operationalise through biological measurements, which are very often missing.

Estimates from EHRs are sensitive to the code lists and that our findings need to be interpreted with caution[18], however, our code list included only a few relevant items and is not sensitive to misclassification. For BMI and cholesterol, we categorise and include a "missing" category, which can be problematic, but allows us to observe the associations with T2DM conversion. Our risk prediction model did not attempt to include and reaffirm all known drivers of diabetes, but we primarily aimed to examine the role of socio-economic drivers and lifestyle factors, along with depression (potentially actionable and important comorbidity for T2DM [19]), and a proxy for "overall health". Alcohol intake was not included in the model, since the quality of recording such information in UK primary care is rather poor [20]. We also decided not to use medication for two reasons: first, we would need to capture and organise everything to a patient (and the relevant volumes), which is a tremendous amount of work, with no clear link to conversion as far as we know; secondly, and more importantly, including

For peer review only - http://bmjopen.bmj.com/site/about/guidelines.xhtml

treatment in our model would probably introduce unmeasured confounding, with treatments being associated to conversion when the underlying conditions and the health of the patient are the driving causes.

Our findings suggested the women were at a lower risk of conversion from NDH to T2DM than men. Previous studies have shown that the incidence of diabetes in those diagnosed with prediabetes was higher in women [10]. The difference may be due to different populations studied (two of the three studies were on American Indians and the other was an Australian population). The discrepancies may also be due to the different definition of NDH used [21]. For example in the Australian study which followed up 5,842 participants over 5 years, men categorised as having impaired fasting glucose had a higher incidence of diabetes compared to women (4.0% vs 2.0%), whereas women categorised with impaired glucose tolerance (IGT) had a significantly higher incidence of diabetes than men (4.4% vs. 2.9%) [22].

A review [23] exploring the rates of conversion from IGT to T2DM showed rates ranging from 1.5% per year in Bradford, UK to 7% in Mexicans and Americans. In our study, rates of conversion from NDH to T2DM decreased from 2000 till 2015, with peaks in 2004 and 2011. Since studies in primary care data have suggested that the incidence rates of T2DM has stabilised after 2005, [24] this apparent decrease in conversion rates needs to be interpreted with caution. One possible explanation is changes in the definition of NDH, with different HbA1c ranges used over the study period. Another plausible explanation for the decreasing trends is changes in coding practice, with more people of lower conversion risk being linked with NDH in primary care records. In addition, the peak we observed for 2011 might either be due to the uptake of NHS Health Checks which was introduced in April 2009 and also the WHO recommendation in 2011 to use HbA1c for T2DM diagnosis [25]. A systematic review exploring the trends of prediabetes in South Asians, showed that T2DM was rising but the prevalence of IGT was stable or decreasing. They suggested that this might be due to increased testing for T2DM and also studies have found that fasting plasma glucose was more influenced by obesity than 2-hour glucose testing [26]. It has also been suggested that these decreased trends might be due to a more rapid progression from IGT to T2DM with the IGT state possibly skipping altogether in the disease progression [27]. Studies have also shown a change of NDH to normoglycaemia after lifestyle and drug-based interventions, which might also be a reason for our findings [28 29], as the NICE guidelines have also proposed primary care practitioners to advice patients with NDH on diet and exercise as well as drug interventions with metformin in some cases [30]. We found a crude rate of conversion of NDH to T2DM to be about 7%, where a previous report using CPRD in which prediabetes was defined using

For peer review only - http://bmjopen.bmj.com/site/about/guidelines.xhtml

Fasting glucose levels showed the progression of IFG (Impaired fasting glucose) to diabetes was 6% per year [31].

The prevalence of NDH in Health Survey England analyses showed an increase with age, and it increased from 3% in 16-69 age groups to 30.4% in those aged over 80 years [10]. However, our findings showed the risk of conversion to diabetes from NDH decreased with increasing age and the risk was significantly lower in those aged over 75 years compared to those aged 18-44. Similar associations were shown in The Strong Heart Study which suggested that this might be due to the survival effect in the older adults and the prevalence of obesity being higher in younger adults [32]. An analysis of six prospective studies which explored the predictors of progression from Impaired Glucose Tolerance (IGT) to Non-Insulin Dependent Diabetes Mellitus (NIDDM) found inconsistent relationships with age. In the studies with the highest incidence rates of NIDDM, the progression of NIDDM increased with age in participants diagnosed with IGT at a younger age and decreased with age in participants who were diagnosed with IGT at an older age [33].There was a negative association in those aged over 85 years and the risk of conversion from NDH to T2DM. This negative association may be due to the fact older population may be less likely to be checked for T2DM in primary care [31] or the threshold needed to identify NDH in older adults may need to be reconsidered.

We also found the risk of conversion of NDH increased with increase in BMI. Obesity has been linked to increased prevalence of prediabetes previously [34], however several other studies exploring the progression of prediabetes to T2DM have shown conflicting results with BMI playing a small or non-significant role [33].

We also showed that current smokers were more likely to convert from NDH to T2DM. In the Health Survey England data it was shown that the prevalence of prediabetes was significantly higher in ex-smokers compared to non-smokers [10]. Our results also showed a high cholesterol levels were associated with a reduced risk of developing T2DM. Previous studies to our knowledge have not explored the relation of cholesterol with progression of prediabetes to diabetes. Our findings also indicated that having a 1-2 Charlson comorbidity score increased the risk of progression to T2DM; however, we were not able to distinguish which co-morbidities were linked to progression from NDH to T2DM.

Socioeconomic inequalities exist in health care, a fact that has been summarised by Hart's inverse care law which suggests that those in most need of health care are those least likely to receive it [35]. Our

For peer review only - http://bmjopen.bmj.com/site/about/guidelines.xhtml

findings that the risk of conversion of NDH to T2DM was higher in those of lower socioeconomic status has not been reported previously, to our knowledge. Although a previous report on NDH by Public Health England using the Health Survey England data showed that there was no significant difference in the prevalence of NDH by quintile of deprivation, the study did not explore the risk of conversion from NDH to T2DM [10]. Our results align with previous findings which have suggested that impaired glucose regulation (IGR/NDH) and T2DM are more prevalent in those with low socioeconomic status [6 7].

Conclusions

Over the study period, the conversion rate of NDH to T2DM was, on average, 7% within a year. However, there was a large reduction in that rate over time, which should be attributed to changes in coding practices and in the definition of NDH, rather than a reduction in the incidence of T2DM. The key predictors in the progression of NDH to T2DM were age, increased BMI and lower socioeconomic status. It would be interesting to examine the population trends of progression from NDH to T2DM following the introduction of the National Diabetes Prevention Programme, a behavioural intervention programme targeted at people with a high risk of developing T2DM [9].

For peer review only

Table 1: Prevalence and Incidence of NDH

	Prevalence			Incidence		
Year	Numerator	Denominator	%	Numerator	Denominator	%
2000	2809	3,784,862	0.07	750	3,782,803	0.02
2001	4065	3,825,769	0.11	1256	3,822,960	0.03
2002	6627	3,868,575	0.17	2562	3,864,510	0.07
2003	10,790	3,905,077	0.28	4163	3,898,450	0.11
2004	16,687	3,957,556	0.42	5897	3,946,766	0.15
2005	23,989	3,996,114	0.60	7302	3,979,427	0.18
2006	29,805	4,029,795	0.74	5816	4,005,806	0.15
2007	35,730	4,074,123	0.88	5925	4,044,318	0.15
2008	41,930	4,130,943	1.02	6200	4,095,213	0.15
2009	48,116	4,191,018	1.15	6186	4,149,088	0.15
2010	52,891	4,245,410	1.25	4775	4,197,294	0.11
2011	57,556	4,283,200	1.34	4665	4,230,309	0.11
2012	61,787	4,335,322	1.43	4231	4,277,766	0.10
2013	68,376	4,383,749	1.56	6589	4,321,962	0.15
2014	74,423	4,446,718	1.67	6047	4,378,342	0.14
2015	83,652	4,528,613	1.85	9229	4,454,190	0.21

Note: Year 2000 defined as 01st April 2000 till 31st March 2001 and other years defined similarly

For peer review only

For peer review only - http://bmjopen.bmj.com/site/about/guidelines.xhtml

Table 2: Cumulative frequency of conversion from NDH to T2DM from 2000 to 2015

	**Within 1 month**				**Within 3 months**				**Within 6 months**				**Within 1 year**			
**Year**	**N remaining unconverted**	**N converted**	**N censored**	**Cum % converted**	**N remaining unconverted**	**N converted**	**N censored**	**Cum % converted**	**N remaining unconverted**	**N converted**	**N censored**	**Cum % converted**	**N remaining unconverted**	**N converted**	**N censored**	**Cum % converted**
**2000**	887	19	1	2.10	870	13	4	3.53	854	15	1	5.20	818	25	11	7.99
**2001**	1460	35	0	2.34	1433	26	1	4.08	1397	29	7	6.03	1320	58	19	9.96
**2002**	2922	72	2	2.40	2863	55	4	4.24	2803	47	13	5.82	2650	126	27	10.07
**2003**	4793	115	5	2.34	4655	125	13	4.89	4538	85	32	6.63	4276	183	79	10.43
**2004**	7076	184	6	2.53	6907	151	18	4.62	6698	160	49	6.83	6370	241	87	10.21
**2005**	8832	185	7	2.05	8660	152	20	3.74	8479	132	49	5.21	8007	335	137	8.99
**2006**	8561	193	4	2.20	8389	149	23	3.91	8194	140	55	5.52	7743	319	132	9.23
**2007**	9240	192	14	2.03	9073	144	23	3.56	8912	130	31	4.95	8472	317	123	8.35
**2008**	10243	179	10	1.72	10046	172	25	3.37	9871	114	61	4.47	9391	370	110	8.07
**2009**	10923	191	8	1.72	10721	185	17	3.38	10553	123	45	4.49	10100	319	134	7.40
**2010**	9991	189	4	1.86	9828	146	17	3.29	9686	107	35	4.35	9279	291	116	7.24
**2011**	9973	163	6	1.61	9792	161	20	3.20	9628	126	38	4.45	9181	309	138	7.53
**2012**	10057	162	5	1.58	9912	130	15	2.86	9743	131	38	4.14	9366	274	103	6.85
**2013**	12267	131	17	1.06	12130	110	27	1.94	11963	115	52	2.88	11537	264	162	5.03
**2014**	11318	85	14	0.74	11214	71	33	1.37	11061	92	61	2.18	10717	209	135	4.04
**2015**	12832	81	1080	0.60	10111	85	2636	1.34	6716	72	3323	2.18				

For peer review only

Table 2 contd: Cumulative frequency of conversion from NDH to T2DM from 2000 to 2015

	Within 2 years				Within 3 years				Within 4 years				Within 5 years			
**Year**	**N remaining unconverted**	**N converted**	**N censored**	**Cum % converted**	**N remaining unconverted**	**N converted**	**N censored**	**Cum % converted**	**N remaining unconverted**	**N converted**	**N censored**	**Cum % converted**	**N remaining unconverted**	**N converted**	**N censored**	**Cum % converted**
**2000**	734	62	22	15.06	634	68	32	23.10	545	57	32	30.20	456	60	29	38.09
**2001**	1160	103	57	17.14	971	135	54	27.01	827	94	50	34.26	694	76	57	40.52
**2002**	2283	256	111	18.95	1973	210	100	26.57	1674	198	101	34.13	1377	191	106	41.89
**2003**	3647	437	192	19.80	3105	359	183	27.89	2672	272	161	34.38	2305	228	139	40.13
**2004**	5490	590	290	18.72	4726	471	293	25.88	4086	384	256	32.07	3533	325	228	37.63
**2005**	6939	711	357	17.25	6025	577	337	24.30	5275	459	291	30.21	4650	406	219	35.70
**2006**	6741	700	302	17.60	5841	638	262	25.55	5076	467	298	31.66	4468	341	267	36.37
**2007**	7328	829	315	17.49	6385	643	300	24.88	5612	484	289	30.71	4959	379	274	35.50
**2008**	8176	836	379	16.42	7247	602	327	22.70	6473	474	300	27.86	5763	421	289	32.66
**2009**	9059	708	333	14.00	8049	621	389	20.02	7229	500	320	25.09	6597	344	288	28.73
**2010**	8324	616	339	13.51	7427	587	310	19.73	6712	440	275	24.57	6186	306	220	28.07
**2011**	8091	773	317	15.46	7303	473	315	20.50	6703	342	258	24.29	0	137	6566	27.32
**2012**	8467	537	362	12.30	7769	366	332	16.17								
**2013**	10625	487	425	9.12												

For peer review only

For peer review only - http://bmjopen.bmj.com/site/about/guidelines.xhtml

Table 3: Characteristics of the cohort

	All	Males	Females
**N**	141,272	73,903 (52.3)	67,369 (47.7)
**Age (years)**	63.2±13.4	62.8±12.4	63.6±14.5
**Age group**	**Count (%)**		
**18-44**	12,896 (9.1)	5619 (7.6)	7277 (10.8)
**45-54**	22,717 (16.1)	12,934 (17.5)	9783 (14.5)
**55-64**	36,790 (26.0)	21,127 (28.6)	15,663 (23.3)
**65-74**	39,178 (27.7)	21,042 (28.5)	18,136 (26.9)
**75-84**	23,801 (16.9)	11,019 (14.9)	12,782 (19.0)
**>=85**	5890 (4.2)	2162 (2.9)	3728 (5.5)
**Smoking Status**	**Count (%)**		
**Current**	21,088 (14.9)	11,352 (15.4)	9736 (14.5)
**Ex**	46,301 (32.8)	27,979 (37.9)	18,322 (27.2)
**Never**	27,834 (19.7)	12,046 (16.3)	15,788 (23.4)
**Missing**	46,049 (32.6)	22,526 (30.5)	23,523 (34.9)
**BMI Categories (kg/m²)**	**Count (%)**		
**<18.5**	628 (0.4)	153 (0.2)	475 (0.7)
**18.5-25**	11,553 (8.2)	5504 (7.5)	6049 (9.0)
**25-30**	27,523 (19.5)	16,686 (22.6)	10,837 (16.1)
**>=30**	42,456 (30.1)	21,189 (28.7)	21,267 (31.6)
**Missing**	59,112 (41.8)	30,371 (41.1)	28,741 (42.7)
**Cholesterol (%)**	**Count (%)**		
**<3**	1538 (1.1)	1203 (1.6)	336 (0.5)
**3 to 4**	12,668 (9.0)	8814 (11.9)	3859 (5.7)
**4 to 5**	29,204 (20.7)	17,170 (23.2)	12,041 (17.9)
**5 to 6**	28,554 (20.2)	14,889 (20.1)	13,670 (20.3)
**>=6**	22,818 (16.2)	9844 (13.3)	12,979 (19.3)
**Missing**	46,490 (32.9)	22,002 (29.8)	24,513 (36.4)
**Depression**	26,064 (18.5)	9724 (13.2)	16,340 (24.3)
**CCI Score**	**Count (%)**		
**None**	120,158 (85.1)	63,571 (86.0)	56,587 (84.0)
**1 to 2**	20,912 (14.8)	10,215 (13.8)	10,697 (15.9)
**3 to 4**	142 (0.1)	85 (0.1)	57 (0.1)
**>4**	60 (0.04)	32 (0.04)	28 (0.04)
**Patient level deprivation Index (2010 IMD score)**			**Count (%)**
**Quintile 1(Most Affluent)**	12,854 (9.1)	7034 (9.5)	5820 (8.6)
**Quintile 2**	13,617 (9.6)	7368 (10.0)	6249 (9.3)
**Quintile 3**	12,882 (9.1)	6692 (9.1)	6190 (9.2)
**Quintile 4**	12,816 (9.1)	6514 (8.8)	6302 (9.4)
**Quintile 5(Least Affluent)**	9866 (7.0)	4780 (6.5)	5086 (7.6)
**Missing**	79,237 (56.1)	41,515 (56.2)	37,722 (56.0)

For peer review only - http://bmjopen.bmj.com/site/about/guidelines.xhtml

Table 4: Conversion from at risk of diabetes (NDH) to T2DM

Time taken to convert from at risk to T2Diabetes	Numerator (total number diagnosed with T2D)	Denominator (total number with NDH)	%	% Change
Within 1 month	2,176	134,734	1.62	
Within 3months	4,051	134,734	3.01	1.39
Within 6months	5,669	134,734	4.21	1.20
Within 1 year	9,369	134,734	6.95	2.75
Within 2 years	17,216	134,734	12.78	5.82
Within 3 years	23,168	134,734	17.20	4.42
Within 4 years	27,490	134,734	20.40	3.21
Within 5 years	30,704	134,734	22.79	2.39

For peer review only

Table 5: Cox proportional hazard models exploring time to conversion from NDH to T2DM for patients by baseline characteristics

	HR (95% CI)	p value
**Males**	Ref	
**Females**	0.97 (0.95 to 0.99)	0.009
**Age Group (Years)**		
**18-44**	Ref	
**45-54**	1.20 (1.15 to 1.25)	<0.001
**55-64**	1.10 (1.06 to 1.14)	<0.001
**65-74**	1.03 (0.99 to 1.07)	0.13
**75-84**	0.86 (0.82 to 0.90)	<0.001
**>=85**	0.65 (0.60 to 0.71)	<0.001
**Cholesterol categories (%)**		
**<3**	1.04 (0.95 to 1.16)	0.391
**3 to 4**	1.03 (0.99 to 1.07)	0.165
**4 to 5**	Ref	
**5 to 6**	0.94 (0.92 to 0.98)	0.001
**>=6**	0.92 (0.89 to 0.95)	<0.001
**Missing**	0.91 (0.89 to 0.94)	<0.001
**Smoking Status**		
**Non smoker**	Ref	
**Current Smoker**	1.07 (1.03 to 1.11)	<0.001
**Ex- smoker**	0.98 (0.96 to 1.01)	0.312
**missing**	0.98 (0.95 to 1.02)	0.338
**BMI Categories(kg/m^2)**		
**<18.5**	0.59 (0.44 to 0.78)	<0.001
**18.5-25**	Ref	
**25-30**	1.40 (1.33 to 1.48)	<0.001
**>=30**	2.02 (1.92 to 2.13)	<0.001
**Missing**	1.44 (1.37 to 1.52)	<0.001
**Depression**	1.10 (1.07 to 1.13)	<0.001
**CCI Score**		
**None**	Ref	
**1 to 2**	1.11 (1.08 to 1.15)	<0.001
**3 to 4**	0.98 (0.68 to 1.43)	0.934
**>4**	1.67 (0.99 to 2.81)	0.057
**Patient level Deprivation Index**		
**Quintile 1(Most Affluent)**	Ref	
**Quintile 2**	1.08 (1.03 to 1.13)	0.002
**Quintile 3**	1.03 (0.98 to 1.08)	0.237
**Quintile 4**	1.12 (1.07 to 1.18)	<0.001
**Quintile 5(Least Affluent)**	1.17 (1.11 to 1.24)	<0.001
**Missing**	1.13 (1.09 to 1.18)	<0.001
**Year trend**	0.94 (0.94 to 0.95)	<0.001

For peer review only

Figure 1: Flow diagram of patient inclusions

Figure 2: Cumulative conversion of NDH to T2DM diabetes from 2000 till 2015

For peer review only

For peer review only - http://bmjopen.bmj.com/site/about/guidelines.xhtml

References

1. Worldwide trends in diabetes since 1980: a pooled analysis of 751 population-based studies with 4.4 million participants. *The Lancet* 2016;387(10027):1513-30. doi: 10.1016/S0140-6736(16)00618-8
2. Hudson H. The NHS Heath Check screening and non-diabetic hyperglycaemia. *Practice Nursing* 2016;27(10):473-80. doi: 10.12968/pnur.2016.27.10.473
3. Defronzo RA. Banting Lecture. From the triumvirate to the ominous octet: a new paradigm for the treatment of type 2 diabetes mellitus. *Diabetes* 2009;58(4):773-95. doi: 10.2337/db09-9028
4. Federation ID. IDF Diabetes Atlas. 8th Edition ed: Brussels, Belgium, 2017.
5. NHS. NHS RightCare Pathway: Diabetes. In: England N, ed., 2018:1-21.
6. Moody A, Cowley G, Ng Fat L, et al. Social inequalities in prevalence of diagnosed and undiagnosed diabetes and impaired glucose regulation in participants in the Health Surveys for England series. *BMJ open* 2016;6(2) doi: 10.1136/bmjopen-2015-010155
7. Mainous AG, 3rd, Tanner RJ, Baker R, et al. Prevalence of prediabetes in England from 2003 to 2011: population-based, cross-sectional study. *BMJ open* 2014;4(6):e005002. doi: 10.1136/bmjopen-2014-005002 [published Online First: 2014/06/11]
8. Barry E, Roberts S, Oke J, et al. Efficacy and effectiveness of screen and treat policies in prevention of type 2 diabetes: systematic review and meta-analysis of screening tests and interventions. *BMJ* 2017;356:i6538. doi: 10.1136/bmj.i6538
9. England PHEDUN. NHS Diabetes Prevention Programme, NHSDPP overview and FAQ *NHS England Publications* 2016(Gateway Reference 05728)
10. England PH. NHS Diabetes Prevention Programm (NHS DPP) Non-diabetic hyperglycaemia. In: (NCVIN) NCIN, ed. London: PHE publications, 2015.
11. Herrett E, Gallagher AM, Bhaskaran K, et al. Data Resource Profile: Clinical Practice Research Datalink (CPRD). *International journal of epidemiology* 2015;44(3):827-36. doi: 10.1093/ije/dyv098 [published Online First: 2015/06/08]
12. McLennan; D, Barnes; H, Noble; M, et al. The English indices of deprivation 2010. Online First: Oxford Institute of Social Policy, University of Oxford, 2011.
13. Springate DA, Kontopantelis E, Ashcroft DM, et al. ClinicalCodes: An Online Clinical Codes Repository to Improve the Validity and Reproducibility of Research Using Electronic Medical Records. *PLoS ONE* 2014;9(6):e99825. doi: 10.1371/journal.pone.0099825
14. Olier I, Springate DA, Ashcroft DM, et al. Modelling Conditions and Health Care Processes in Electronic Health Records: An Application to Severe Mental Illness with the Clinical Practice Research Datalink. *PLoS One* 2016;11(2):e0146715. doi: 10.1371/journal.pone.0146715 [published Online First: 2016/02/27]
15. Khan NF, Perera R, Harper S, et al. Adaptation and validation of the Charlson Index for Read/OXMIS coded databases. *BMC family practice* 2010;11:1. doi: 10.1186/1471-2296-11-1 [published Online First: 2010/01/07]
16. Charlson ME, Pompei P, Ales KL, et al. A new method of classifying prognostic comorbidity in longitudinal studies: development and validation. *Journal of chronic diseases* 1987;40(5):373-83. [published Online First: 1987/01/01]
17. CPRD @ Cambridge – Codes Lists Version 1.1 October 2018 [Available from: https://www.phpc.cam.ac.uk/pcu/cprd_cam/codelists/v11/ accessed 26/11/2018 2018.
18. Tate AR, Dungey S, Glew S, et al. Quality of recording of diabetes in the UK: how does the GP's method of coding clinical data affect incidence estimates? Cross-sectional study using the CPRD database. *BMJ open* 2017;7(1) doi: 10.1136/bmjopen-2016-012905
19. de Groot M, Anderson R, Freedland KE, et al. Association of Depression and Diabetes Complications: A Meta-Analysis. *Psychosomatic Medicine* 2001;63(4):619-30.
20. Nowakowska M, Zghebi SS, Ashcroft DM, et al. The comorbidity burden of type 2 diabetes mellitus: patterns, clusters and predictions from a large English primary care cohort. *BMC*

For peer review only - http://bmjopen.bmj.com/site/about/guidelines.xhtml

medicine 2019;17(1):145. doi: 10.1186/s12916-019-1373-y [published Online First: 2019/07/28]

21. Morris DH, Khunti K, Achana F, et al. Progression rates from HbA1c 6.0-6.4% and other prediabetes definitions to type 2 diabetes: a meta-analysis. *Diabetologia* 2013;56(7):1489-93. doi: 10.1007/s00125-013-2902-4 [published Online First: 2013/04/16]
22. Magliano DJ, Barr EL, Zimmet PZ, et al. Glucose indices, health behaviors, and incidence of diabetes in Australia: the Australian Diabetes, Obesity and Lifestyle Study. *Diabetes care* 2008;31(2):267-72. doi: 10.2337/dc07-0912 [published Online First: 2007/11/09]
23. Harris MI. Impaired glucose tolerance--prevalence and conversion to NIDDM. *Diabetic medicine : a journal of the British Diabetic Association* 1996;13(3 Suppl 2):S9-11. [published Online First: 1996/01/01]
24. Sharma M, Nazareth I, Petersen I. Trends in incidence, prevalence and prescribing in type 2 diabetes mellitus between 2000 and 2013 in primary care: a retrospective cohort study. *BMJ open* 2016;6(1):e010210. doi: 10.1136/bmjopen-2015-010210 [published Online First: 2016/01/16]
25. (WHO) WHO. Use of glycated haemoglobin (HbA1c) in the diagnosis of diabetes mellitus, 2011:25.
26. Katikireddi SV, Morling JR, Bhopal R. Is there a divergence in time trends in the prevalence of impaired glucose tolerance and diabetes? A systematic review in South Asian populations. *International journal of epidemiology* 2011;40(6):1542-53. doi: 10.1093/ije/dyr159
27. Mohan V, Deepa M, Deepa R, et al. Secular trends in the prevalence of diabetes and impaired glucose tolerance in urban South India—the Chennai Urban Rural Epidemiology Study (CURES-17). *Diabetologia* 2006;49(6):1175-78. doi: 10.1007/s00125-006-0219-2
28. Tabák AG, Herder C, Rathmann W, et al. Prediabetes: A high-risk state for developing diabetes. *Lancet* 2012;379(9833):2279-90. doi: 10.1016/S0140-6736(12)60283-9
29. DeFronzo RA, Tripathy D, Schwenke DC, et al. Pioglitazone for diabetes prevention in impaired glucose tolerance. *The New England journal of medicine* 2011;364(12):1104-15. doi: 10.1056/NEJMoa1010949 [published Online First: 2011/03/25]
30. (NICE) NIfHaCE. Type 2 diabetes: prevention in people at high risk, 2017:1-42.
31. Hong J-L, McNeill AM, He J, et al. Identification of impaired fasting glucose, healthcare utilization and progression to diabetes in the UK using the Clinical Practice Research Datalink (CPRD). *Pharmacoepidemiology and Drug Safety* 2016;25(12):1375-86. doi: doi:10.1002/pds.4007
32. Wang H, Shara NM, Calhoun D, et al. Incidence rates and predictors of diabetes in those with prediabetes: the Strong Heart Study. *Diabetes/metabolism research and reviews* 2010;26(5):378-85. doi: 10.1002/dmrr.1089
33. Edelstein SL, Knowler WC, Bain RP, et al. Predictors of progression from impaired glucose tolerance to NIDDM: an analysis of six prospective studies. *Diabetes* 1997;46(4):701-10.
34. Ben Haider NY, Ziyab AH. Prevalence of prediabetes and its association with obesity among college students in Kuwait: A cross-sectional study. *Diabetes Research and Clinical Practice* 2016;119:71-74. doi: https://doi.org/10.1016/j.diabres.2016.07.001
35. Tudor Hart J. THE INVERSE CARE LAW. *The Lancet* 1971;297(7696):405-12. doi: 10.1016/S0140-6736(71)92410-X

For peer review only

For peer review only - http://bmjopen.bmj.com/site/about/guidelines.xhtml

Acknowledgments

This manuscript is independent research funded by the National Institute for Health Research (Health Services and Delivery Research, 16/48/07 – Evaluating the NHS Diabetes Prevention Programme (NHS DPP): the DIPLOMA research programme (Diabetes Prevention – Long Term Multimethod Assessment)).

The views and opinions expressed in this manuscript are those of the authors and do not necessarily reflect those of the NHS, the National Institute for Health Research or the Department of Health and Social Care.

Copyright

The Corresponding Author has the right to grant on behalf of all authors and does grant on behalf of all authors, a worldwide licence to the Publishers and its licensees in perpetuity, in all forms, formats and media (whether known now or created in the future), to i) publish, reproduce, distribute, display and store the Contribution, ii) translate the Contribution into other languages, create adaptations, reprints, include within collections and create summaries, extracts and/or, abstracts of the Contribution, iii) create any other derivative work(s) based on the Contribution, iv) to exploit all subsidiary rights in the Contribution, v) the inclusion of electronic links from the Contribution to third party material where-ever it may be located; and, vi) licence any third party to do any or all of the above.

For peer review only

Declaration of competing interests

National Institute for Health Research (Health Services and Delivery Research, 16/48/07 – Evaluating the NHS Diabetes Prevention Programme (NHS DPP): the DIPLOMA research programme (Diabetes Prevention – Long Term Multimethod Assessment)). Funded the time and facilities of RR. SH contributes for consultancy for Eli Lilly, NovoNordisk, Takeda, Sanofi Aventis, Zealand Pharma, UN-EEG and is also part of the speakers panel for NovoNordisk. No other relationships or activities that could appear to have influenced the submitted work.

Authorship & contributorship

EK & RR designed the study, RR extracted the data from all sources and performed the analyses. RR wrote the manuscript. DR, EH, RM, SRC, SC, WW, SH, MS, PB and EK critically revised the manuscript. RR is the guarantor of this work and, as such, had full access to all the data in the study and takes responsibility for the integrity of the data and the accuracy of the data analysis.

Transparency declaration

RR affirms that this manuscript is an honest, accurate, and transparent account of the study being reported; that no important aspects of the study have been omitted; and that any discrepancies from the study as planned have been explained.

Data sharing

The data used in this study cannot be shared due to licencing restrictions by CPRD.

For peer review only - http://bmjopen.bmj.com/site/about/guidelines.xhtml

Dissemination Declaration

Not applicable

Ethical approval

The protocol for this study received scientific and ethical approval from the Independent Scientific Advisory Committee for CPRD studies (ISAC Protocol 18_101).

For peer review only

For peer review only - http://bmjopen.bmj.com/site/about/guidelines.xhtml

Figure 1: Flow diagram of patient inclusions

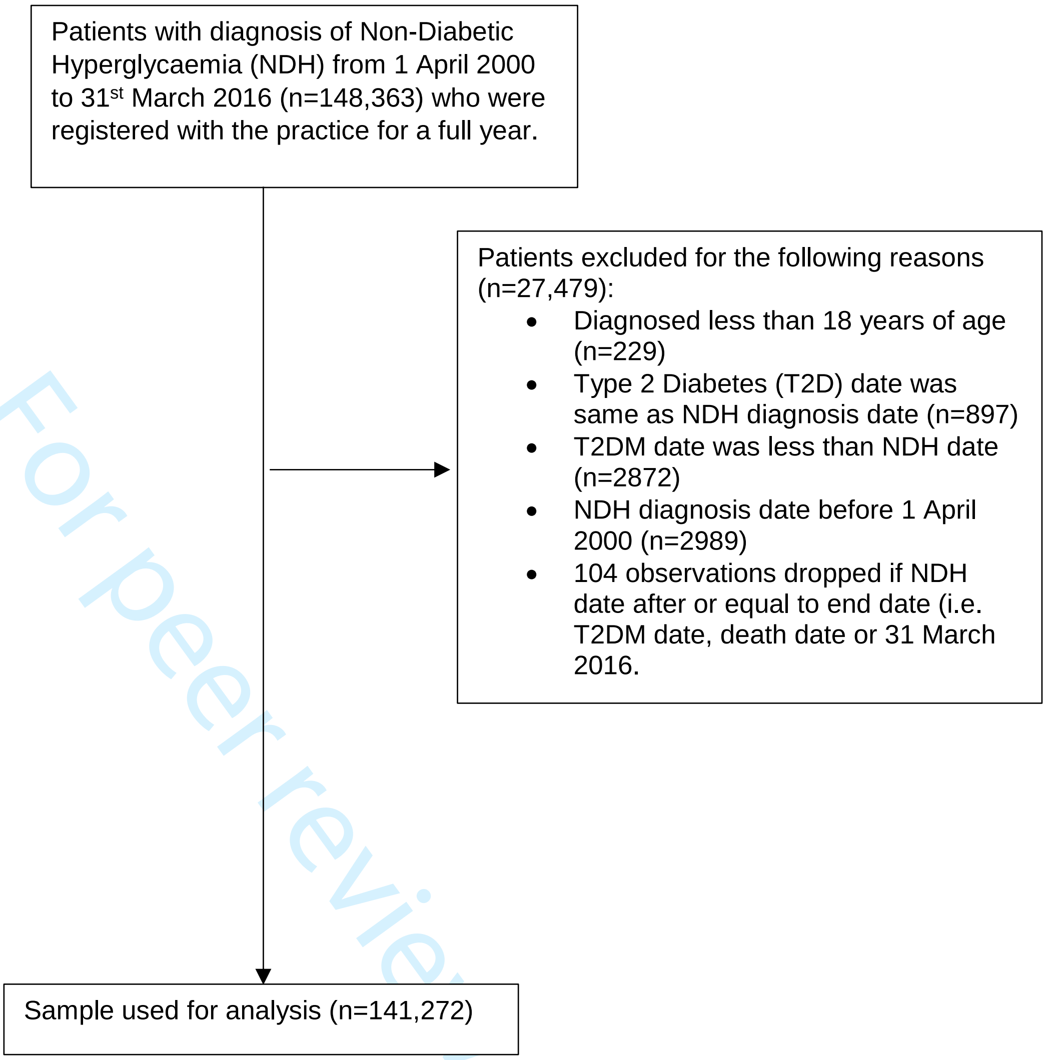

For peer review only

Figure 2: Cumulative conversion of NDH to type-2 diabetes from 2000 till 2015

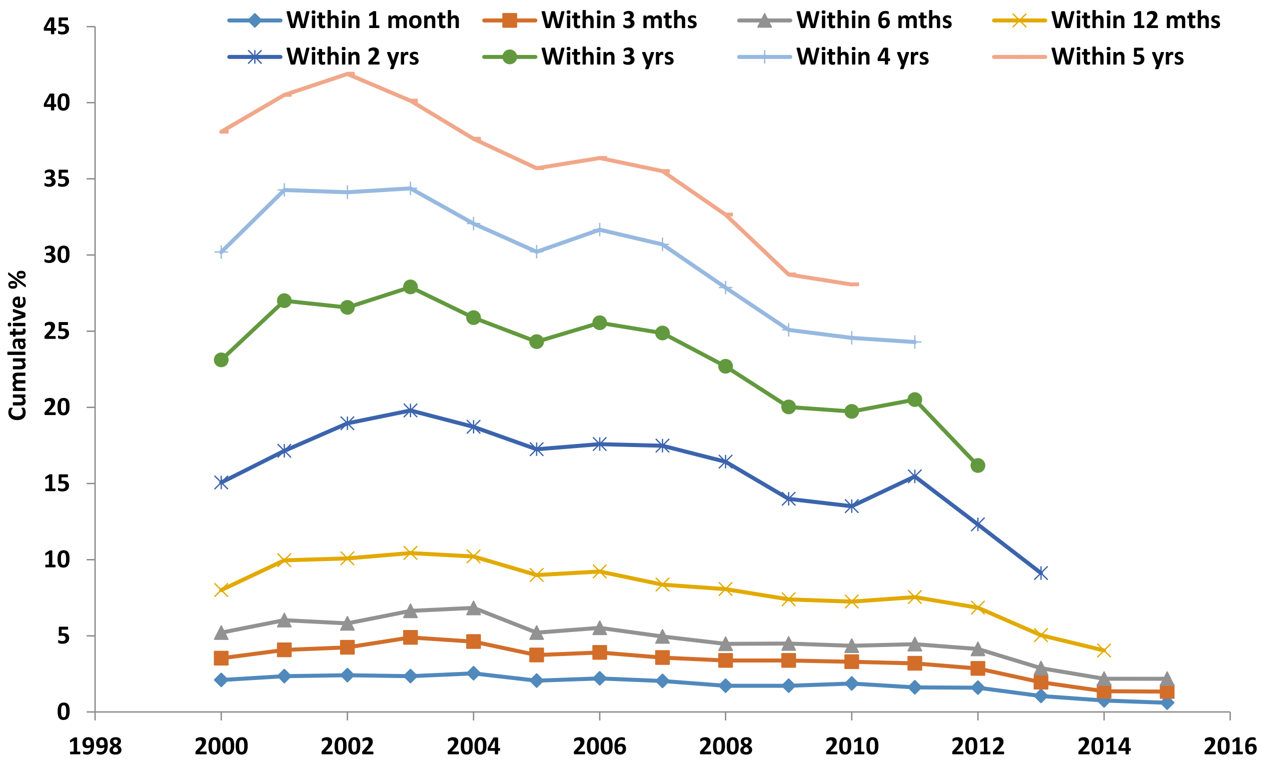

Note: Year 2000 defined as 01st April 2000 till 31st March 2001 and other years defined similarly

review only

Supplementary

Table 1: Read codes used to diagnose Type 2 Diabetes Mellitus

Medcode	Readcode	Description
506	C100112	Non-insulin dependent diabetes mellitus
758	C10F.00	Type 2 diabetes mellitus
1407	C10FJ00	Insulin treated Type 2 diabetes mellitus
4513	C109.00	Non-insulin dependent diabetes mellitus
5884	C109.11	NIDDM - Non-insulin dependent diabetes mellitus
8403	C109700	Non-insulin dependent diabetes mellitus - poor control
12640	C10FC00	Type 2 diabetes mellitus with nephropathy
12736	C10F500	Type 2 diabetes mellitus with gangrene
17262	C109600	Non-insulin-dependent diabetes mellitus with retinopathy
17859	C109.12	Type 2 diabetes mellitus
18143	C109G11	Type II diabetes mellitus with arthropathy
18209	C109012	Type 2 diabetes mellitus with renal complications
18219	C109.13	Type II diabetes mellitus
18264	C109J12	Insulin treated Type II diabetes mellitus
18278	C109J00	Insulin treated Type 2 diabetes mellitus
18390	C10FM00	Type 2 diabetes mellitus with persistent microalbuminuria
18425	C10FB00	Type 2 diabetes mellitus with polyneuropathy
18496	C10F600	Type 2 diabetes mellitus with retinopathy
18777	C10F000	Type 2 diabetes mellitus with renal complications
22884	C10F.11	Type II diabetes mellitus
24458	C109711	Type II diabetes mellitus - poor control
24693	C109G00	Non-insulin dependent diabetes mellitus with arthropathy
24836	C109C12	Type 2 diabetes mellitus with nephropathy
25041	ZC2CA00	Dietary advice for type II diabetes
25591	C10FQ00	Type 2 diabetes mellitus with exudative maculopathy
25627	C10F700	Type 2 diabetes mellitus - poor control
26054	C10FL00	Type 2 diabetes mellitus with persistent proteinuria
29979	C109900	Non-insulin-dependent diabetes mellitus without complication
32627	C10FN00	Type 2 diabetes mellitus with ketoacidosis
34268	C10F200	Type 2 diabetes mellitus with neurological complications
34450	C10FK00	Hyperosmolar non-ketotic state in type 2 diabetes mellitus
34912	C109400	Non-insulin dependent diabetes mellitus with ulcer
35385	C10FH00	Type 2 diabetes mellitus with neuropathic arthropathy
36633	C109K00	Hyperosmolar non-ketotic state in type 2 diabetes mellitus
36695	C10D.00	Diabetes mellitus autosomal dominant type 2
37648	C109J11	Insulin treated non-insulin dependent diabetes mellitus
37806	C10FF00	Type 2 diabetes mellitus with peripheral angiopathy
40401	C109500	Non-insulin dependent diabetes mellitus with gangrene
42762	C109612	Type 2 diabetes mellitus with retinopathy
43227	C10F311	Type II diabetes mellitus with multiple complications
43785	C109D00	Non-insulin dependent diabetes mellitus with hypoglyca coma
44779	C109E12	Type 2 diabetes mellitus with diabetic cataract
44982	C10FE00	Type 2 diabetes mellitus with diabetic cataract
45467	C109B00	Non-insulin dependent diabetes mellitus with polyneuropathy
45913	C109712	Type 2 diabetes mellitus - poor control
45919	C109212	Type 2 diabetes mellitus with neurological complications
46150	C109512	Type 2 diabetes mellitus with gangrene

For peer review only - http://bmjopen.bmj.com/site/about/guidelines.xhtml

Medcode	Readcode	Description
46917	C10FD00	Type 2 diabetes mellitus with hypoglycaemic coma
47315	C10F711	Type II diabetes mellitus - poor control
47321	C10F100	Type 2 diabetes mellitus with ophthalmic complications
47409	C109B11	Type II diabetes mellitus with polyneuropathy
47816	C109H11	Type II diabetes mellitus with neuropathic arthropathy
47954	C10F900	Type 2 diabetes mellitus without complication
48192	C109E11	Type II diabetes mellitus with diabetic cataract
49074	C10F400	Type 2 diabetes mellitus with ulcer
49655	C10F611	Type II diabetes mellitus with retinopathy
49869	C109G12	Type 2 diabetes mellitus with arthropathy
50225	C109011	Type II diabetes mellitus with renal complications
50429	C109100	Non-insulin-dependent diabetes mellitus with ophthalm comps
50527	C10FB11	Type II diabetes mellitus with polyneuropathy
50609	L180600	Pre-existing diabetes mellitus, non-insulin-dependent
50813	C109A11	Type II diabetes mellitus with mononeuropathy
51756	C10FP00	Type 2 diabetes mellitus with ketoacidotic coma
52303	C109000	Non-insulin-dependent diabetes mellitus with renal comps
53392	C10F911	Type II diabetes mellitus without complication
54899	C109F11	Type II diabetes mellitus with peripheral angiopathy
55075	C109411	Type II diabetes mellitus with ulcer
55842	C109200	Non-insulin-dependent diabetes mellitus with neuro comps
56268	C109D11	Type II diabetes mellitus with hypoglycaemic coma
56803	C107400	NIDDM with peripheral circulatory disorder
57278	C10F011	Type II diabetes mellitus with renal complications
58604	C109611	Type II diabetes mellitus with retinopathy
59253	C10FG00	Type 2 diabetes mellitus with arthropathy
59365	C109C00	Non-insulin dependent diabetes mellitus with nephropathy
59725	C109111	Type II diabetes mellitus with ophthalmic complications
60699	C109F12	Type 2 diabetes mellitus with peripheral angiopathy
60796	C10FL11	Type II diabetes mellitus with persistent proteinuria
61071	C109D12	Type 2 diabetes mellitus with hypoglycaemic coma
62107	C109511	Type II diabetes mellitus with gangrene
62146	C109300	Non-insulin-dependent diabetes mellitus with multiple comps
62674	C10FA00	Type 2 diabetes mellitus with mononeuropathy
63690	C10FR00	Type 2 diabetes mellitus with gastroparesis
64571	C109C11	Type II diabetes mellitus with nephropathy
64668	C10FJ11	Insulin treated Type II diabetes mellitus
65267	C10F300	Type 2 diabetes mellitus with multiple complications
65704	C109412	Type 2 diabetes mellitus with ulcer
66965	C109H12	Type 2 diabetes mellitus with neuropathic arthropathy
67905	C109211	Type II diabetes mellitus with neurological complications
69278	C109E00	Non-insulin depend diabetes mellitus with diabetic cataract
70316	C109112	Type 2 diabetes mellitus with ophthalmic complications
72320	C109A00	Non-insulin dependent diabetes mellitus with mononeuropathy
83532	66Ao.00	Diabetes type 2 review
85991	C10FM11	Type II diabetes mellitus with persistent microalbuminuria
91646	C10F411	Type II diabetes mellitus with ulcer
93727	C10FE11	Type II diabetes mellitus with diabetic cataract
95351	C10FA11	Type II diabetes mellitus with mononeuropathy

For peer review only - http://bmjopen.bmj.com/site/about/guidelines.xhtml

Medcode	Readcode	Description
98616	C10F211	Type II diabetes mellitus with neurological complications
98723	C10FD11	Type II diabetes mellitus with hypoglycaemic coma
100964	C10F111	Type II diabetes mellitus with ophthalmic complications
101801	66At100	Type II diabetic dietary review
102201	C10FC11	Type II diabetes mellitus with nephropathy
102611	66At111	Type 2 diabetic dietary review
103902	C10FG11	Type II diabetes mellitus with arthropathy
104323	C10F511	Type II diabetes mellitus with gangrene
104639	C10FF11	Type II diabetes mellitus with peripheral angiopathy
105784	C109912	Type 2 diabetes mellitus without complication
106061	C10FP11	Type II diabetes mellitus with ketoacidotic coma
106528	C10FN11	Type II diabetes mellitus with ketoacidosis
107701	C10FK11	Hyperosmolar non-ketotic state in type II diabetes mellitus
108005	C109312	Type 2 diabetes mellitus with multiple complications
109103	C109911	Type II diabetes mellitus without complication
109197	C10FH11	Type II diabetes mellitus with neuropathic arthropathy
109865	C109B12	Type 2 diabetes mellitus with polyneuropathy
111798	C10FQ11	Type II diabetes mellitus with exudative maculopathy

For peer review only

For peer review only - http://bmjopen.bmj.com/site/about/guidelines.xhtml

STROBE Statement—Checklist of items that should be included in reports of ***cohort studies***

	Item No	Recommendation	Page No
**Title and abstract**	1	(*a*) Indicate the study's design with a commonly used term in the title or the abstract	1-2
		(*b*) Provide in the abstract an informative and balanced summary of what was done and what was found	2
**Introduction**			
Background/rationale	2	Explain the scientific background and rationale for the investigation being reported	3
Objectives	3	State specific objectives, including any prespecified hypotheses	3-4
**Methods**			
Study design	4	Present key elements of study design early in the paper	4
Setting	5	Describe the setting, locations, and relevant dates, including periods of recruitment, exposure, follow-up, and data collection	4
Participants	6	(*a*) Give the eligibility criteria, and the sources and methods of selection of participants. Describe methods of follow-up	4
		(*b*) For matched studies, give matching criteria and number of exposed and unexposed	
Variables	7	Clearly define all outcomes, exposures, predictors, potential confounders, and effect modifiers. Give diagnostic criteria, if applicable	5
Data sources/ measurement	8*	For each variable of interest, give sources of data and details of methods of assessment (measurement). Describe comparability of assessment methods if there is more than one group	5
Bias	9	Describe any efforts to address potential sources of bias	5
Study size	10	Explain how the study size was arrived at	4
Quantitative variables	11	Explain how quantitative variables were handled in the analyses. If applicable, describe which groupings were chosen and why	5-6
Statistical methods	12	(*a*) Describe all statistical methods, including those used to control for confounding	7
		(*b*) Describe any methods used to examine subgroups and interactions	
		(*c*) Explain how missing data were addressed	
		(*d*) If applicable, explain how loss to follow-up was addressed	
		(*e*) Describe any sensitivity analyses	
**Results**			
Participants	13*	(a) Report numbers of individuals at each stage of study—eg numbers potentially eligible, examined for eligibility, confirmed eligible, included in the study, completing follow-up, and analysed	5-6 Figure 1 (PDF)
		(b) Give reasons for non-participation at each stage	
		(c) Consider use of a flow diagram	
Descriptive data	14*	(a) Give characteristics of study participants (eg demographic, clinical, social) and information on exposures and potential confounders	12 Table 3 (Page 15)
		(b) Indicate number of participants with missing data for each variable of interest	
		(c) Summarise follow-up time (eg, average and total amount)	

For peer review only

For peer review only - http://bmjopen.bmj.com/site/about/guidelines.xhtml

Outcome data	15*	Report numbers of outcome events or summary measures over time	5-6

For peer review only - http://bmjopen.bmj.com/site/about/guidelines.xhtml

Main results	16	(*a*) Give unadjusted estimates and, if applicable, confounder-adjusted estimates and their precision (eg, 95% confidence interval). Make clear which confounders were adjusted for and why they were included<br>(*b*) Report category boundaries when continuous variables were categorized<br>(*c*) If relevant, consider translating estimates of relative risk into absolute risk for a meaningful time period	7-16
Other analyses	17	Report other analyses done—eg analyses of subgroups and interactions, and sensitivity analyses	
**Discussion**			
Key results	18	Summarise key results with reference to study objectives	17
Limitations	19	Discuss limitations of the study, taking into account sources of potential bias or imprecision. Discuss both direction and magnitude of any potential bias	17
Interpretation	20	Give a cautious overall interpretation of results considering objectives, limitations, multiplicity of analyses, results from similar studies, and other relevant evidence	20
Generalisability	21	Discuss the generalisability (external validity) of the study results	17
**Other information**			
Funding	22	Give the source of funding and the role of the funders for the present study and, if applicable, for the original study on which the present article is based	24

*Give information separately for exposed and unexposed groups.

Note: An Explanation and Elaboration article discusses each checklist item and gives methodological background and published examples of transparent reporting. The STROBE checklist is best used in conjunction with this article (freely available on the Web sites of PLoS Medicine at http://www.plosmedicine.org/, Annals of Internal Medicine at http://www.annals.org/, and Epidemiology at http://www.epidem.com/). Information on the STROBE Initiative is available at http://www.strobe-statement.org.

For peer review only

For peer review only - http://bmjopen.bmj.com/site/about/guidelines.xhtml
